# The socio-economic determinants of multimorbidity among the elderly population in Trinidad and Tobago

**DOI:** 10.1371/journal.pone.0237307

**Published:** 2020-09-11

**Authors:** Althea La Foucade, Gershwin Davis, Nelleen Baboolal, Don Bethelmie, Christine Laptiste, Haleema Ali-Sisbane, Karl Theodore

**Affiliations:** 1 HEU, Centre for Health Economics, The University of the West Indies, St. Augustine Campus, St. Augustine, Trinidad; 2 The Department of Paraclinical Sciences, Faculty of Medical Sciences, The University of the West Indies, St. Augustine Campus, Eric Williams Medical Sciences Complex, St. Augustine, Trinidad; 3 Faculty of Medical Sciences, The University of the West Indies, St. Augustine Campus, Eric Williams Medical Sciences Complex, St. Augustine, Trinidad; Leibniz Institute for Prevention Research and Epidemiology BIPS, GERMANY

## Abstract

**Objective:**

To estimate the prevalence of multimorbidity and investigate the socioeconomic factors that are associated with multimorbidity among persons 70 years and older in Trinidad and Tobago.

**Design and methods:**

The data were obtained from a nationally representative comprehensive cross-sectional survey conducted in 2014 among elderly persons in the targeted age group. The prevalence of multimorbidity among the elderly population was estimated. A logit model was utilized to determine the socioeconomic characteristics that are associated with multimorbidity in the elderly.

**Results:**

The results of the study show that multimorbidity in the elderly population is strongly associated with age, ethnicity, lower education, smoking history, no physical activity and being female. An interesting finding is that elderly persons in the richest quintile are in general, more prone to multimorbidity.

**Conclusion:**

The findings suggest that interventions to reduce multimorbidity among the elderly population must encourage greater levels of physical activity, provide education on the risk factors of multimorbidity, and discourage smoking.

## Introduction

Trinidad and Tobago, the southernmost twin-island nation in the Caribbean, is experiencing the phenomenon of an ageing population. According to the 2015 World Population Prospects, the share of the elderly population aged 60 years and older is expected to increase from 14.2% in 2015 to 28.2% in 2050, while the percentage of the elderly aged 80 years and older is projected to increase from 1.6% to 4.6% over the same period [1]. This increase in the share of the elderly population is driven by declining fertility rates and improved life expectancy [2].

Research shows that as persons age there is an increased probability of developing more than one chronic medical conditions [3] or multimorbidity, which is defined as the co-existence or co-occurrence of multiple medical conditions in an individual [4, 5]. Several approaches have been used to measure multimorbidity. Some studies incorporate the use of indices such as the Charlson Comorbidity Index [6], and the Index of Co-existent Disease [7]. Others include the use of structured questionnaires that include data on living conditions and social status, which are then analyzed using various statistical methods [5]. The different approaches have often resulted in large differences in estimates of multimorbidity [8, 9, 10].

Most studies on multimorbidity focus on the elderly population [5] and on high-income countries [11]. In general, these studies have shown that multimorbidity among the elderly population is associated with advanced age, being female, low socioeconomic status and low educational attainment [12, 13]. Other studies of have shown differences in multimorbidity among varied ethnic groups [14]. Multimorbidity studies on low- and middle-income countries have been fewer [11]. In one such study on a low-income country, Khanam et al. [3] report that multimorbidity among the elderly population in Bangladesh was significantly higher among women, illiterates, persons who were single, and persons in the non-poorest quintile. Jerliu et al. [15], in their study on Kosovo, a lower-middle-income country, reported multimorbidity prevalence of 42%, and found that female gender, older age, self-perceived poverty, and inability to access care were linked to multimorbidity among the elderly.

Evidence from both low-income and high-income countries indicate that multimorbidity increases with age [13], is significantly more prevalent among elderly females [3, 12, 13, 15], and increases with lower socioeconomic status (SES) [16]. Arokisamy et al. [17] report that there are strong correlations between SES and the prevalence of multimorbidity, when income, occupation, and deprivation are used as variables of SES. In a meta-analysis review of 24 cross-sectional studies, from a mixture of low- and high-income countries, Pathirana and Jackson [16] found that socioeconomic factors such as lower income, higher deprivation, and decreasing income increased the risk of multimorbidity. However, one of the 24 studies pointed to an associated risk of multimorbidity with increased income. This finding seems to suggest that SES measures such as income may also be positively associated with multimorbidity in some low-income countries [11].

Studies on multimorbidity among elderly populations have reported prevalence ranging from 55% to 98% [18]. Using a population-based cross-sectional study to establish the prevalence of dementia among Singapore residents aged 60 years and over, Picco et al. [19] estimated multimorbidity rates of 65% for elderly persons in the 75–84 age group and 59.8% for elderly persons in the 85+ age group. The overall prevalence of multimorbidity for that study was 51.5%. Salive [20], in a survey of 17 studies on multimorbidity, found that the prevalence of multimorbidity in the elderly population was greater than 50%. Further, he reported that 67% of the Medicare beneficiaries in 2008 experienced multimorbidity, and that prevalence increased with age—from 62% of the elderly who were in the 65–74 age bracket to 81.5% of the elderly in the ≥ 85 age bracket. Likewise, Khanam et al. [3] reported a multimorbidity prevalence of 53.8% in their study on the elderly population of rural Bangladesh.

Multimorbidity is also positively correlated with more hospital admissions and longer inpatient stays [21] and results in more post-operative problems, higher health care costs, and higher mortality rates [22]. Multimorbid patients use more medications on average than other health care consumers, and as a result, they are more likely to suffer adverse drug effects [23]. Additionally, they tend to spend more on health care, use a greater range of health care services and live a lower quality of life [24]. The ageing of the population impacts both the utilization of health care services and health care costs [21, 24, 25], and this has implications for the sustainable financing of these services [26].

With the increasing share of elderly persons in Trinidad and Tobago’s population and the strong correlation between ageing and multiple chronic medical conditions, this study seeks to estimate the prevalence of multimorbidity and to analyze the socioeconomic factors associated with multimorbidity among the elderly population. Several factors such as lifestyle habits, economic status, and geographic characteristics as predictors of multimorbidity are considered.

## Materials and methods

### Survey design and sampling

This study uses a population-based, cross-sectional approach to examine the socioeconomic factors associated with multimorbidity among the elderly population aged 70 years and over in Trinidad and Tobago. The data for this study is derived from a comprehensive nationally representative survey of elderly persons 70 years and older. The survey was conducted in 2014 to estimate the prevalence and the economic cost of dementia in Trinidad and Tobago.

The survey methodology that was used to obtain the data for this study is described in detail by Davis et al. [27]. The total number of persons who were invited to participate in the dementia study was 2,378. Of these, a total of 1,898 agreed to participate in the dementia study, which corresponds to a response rate of 79.8%. Data on the socio-demographic characteristics of the elderly persons who did not participate in the dementia study is not available. Of the 480 who did not participate in the dementia study, 128 were not at home on the day of the interview, 142 refused to take part in the survey, 19 were on vacation, 20 were not interviewed because the building was closed, and 171 did not participate for other than the above. Only geographic location of the elderly persons who did not participate is available.

The questionnaire was pilot tested and revised based on the results of the pilot. Approval for the study was granted by The University of the West Indies, St. Augustine, Faculty of Medical Sciences Ethics Committee, and respondents provided written consent for their participation in the survey.

### Measurement

The survey instrument consisted of cognitive and socioeconomic components. The cognitive component was used to measure dementia, and the socioeconomic component was used to collect information on the socioeconomic characteristics of the elderly population.

The following demographic and lifestyle variables were extracted from the survey questionnaire for the study: gender, age, ethnicity, education, level of physical activity, number of cigarettes smoked in the past. In addition to the lifestyle variables, 5 physical assets variables, 4 financial assets variables, two computer variables, 2 categories of the education variable (Primary, Tertiary), 3 categories of the occupation variable (Professional, Semi-skilled Labourer, Unskilled Labourer) and the Chronic Disease Assistance Programme (CDAP) variable were extracted from the survey questionnaire to develop a proxy variable for income (see Table 4). The outcome variable multimorbidity was developed using 17 binary variables that represented the self-reported chronic medical conditions of the elderly participants.

#### Study variables

The primary outcome variable was the presence of multimorbidity in an elderly participant. A binary dependent variable was constructed and assigned the value 1 if the respondent reported having two or more chronic medical conditions. The value 0 was assigned otherwise. The chronic conditions that required extensive treatment were selected for the primary outcome variable, based on input from key informants from the Faculty of Medical Sciences, The University of the West Indies, as well as the Panamerican Steps Chronic Non-Communicable Disease Risk Factor Survey Final Report on Trinidad and Tobago [28].

The predictor variables for this study were divided into four main categories: social factors, lifestyle habits, economic status and geographic region. The social variables considered were: gender (Male, Female), age, ethnicity (African, East Indian, Mixed African & East Indian, Other). The Other category for the education variable represents technical and vocational training.

The lifestyle variables selected were: level of physical activity (very much physically active, fairly physically active, not very much physically active, not at all physically active), and smoking history, which was dichotomized (Heavy smoker, Light smoker) from a discrete variable that represented the number of cigarettes smoked in the past. A heavy smoker was defined as someone who used to smoke at least 20 cigarettes a day, while a light smoker was defined as someone who used to smoke less than 20 cigarettes a day.

The geographic variable represents the fourteen municipality regions of Trinidad. Point Fortin was merged with Siparia for weighting purposes. Hence the total number of municipalities considered for the study was 13. The non-response rate for the income variable was 71%. Hence, a socioeconomic wealth index was developed using Principal Component Analysis (PCA) as a proxy for income to address the low response rate (29%). Seventeen dichotomous socioeconomic variables were used to generate the proxy variable (Table 3).

### Statistical methods

The prevalence of multimorbidity among the elderly population aged 70 years and over was estimated for Trinidad and Tobago. Prevalence for each category of the following variables: gender, age group (70–74, 75–79, 80–84, 85–89, 90+), ethnicity, level of physical activity, educational attainment (Low, High), economic status (quintile 1, quintile 2, quintile 3, quintile 4, quintile 5), and municipality was also computed without weights. A logistic regression model was used to examine the association between multimorbidity and socioeconomic characteristics of the target population [3, 11, 23]. The list of covariates for selection was guided by previous research conducted on multimorbidity [3, 11, 12, 23] and the available data in the dementia survey [27]. All variables that were statistically significant (p = 0.05) were entered into the multivariate regression analysis. The final list of covariates was gender, age, ethnicity, level of physical activity, smoking history, educational attainment, economic status (proxy for income) and municipality. Regression analyses were conducted using unadjusted and adjusted survey sampling weights. Unweighted and weighted odds ratios and their corresponding 95% confidence intervals were computed to examine the socioeconomic factors associated with multimorbidity. Sample weights for each municipality were constructed from the reciprocal of:

$$w_{i}=\frac{1}{p_{i}}\;,$$

where *w*_*i*_ is the municipality weight, and *p*_*i*_ represents the probability of selecting an elderly person in municipality *i* in 2014, the year of the survey (*i* = 1 *to* 14). The probability of selecting an elderly person in each municipality (*p*_*i*_) was computed using the expression $\frac{s_{i}}{n_{i}}$, where *s*_*i*_ is the number of elderly persons sampled in municipality *i*, and *n*_*i*_ is the estimated population for each municipality in the year of the dementia survey.

Population estimates of elderly persons for each municipality were done for 2014 using extrapolation by means of arithmetic rates of increase using the 2000 and 2011 censuses [29].

#### Testing for principal component analysis

There was insufficient income data from the dementia survey. Consequently, PCA was implemented to construct a proxy variable for income, which was defined as economic status in the study [30]. The Kaiser-Meyer-Olkin (KMO) Test, a measure of sampling adequacy, was used to identify multicollinearity among the socioeconomic variables selected. KMO is used to determine whether the selected variables will factor well based on the correlations and partial correlations. It compares the sizes of the observed correlations coefficients and partial correlations. Common factors produce partial correlations that are small in comparison to the correlation coefficient. A KMO value of 0.72 was obtained, which provides sufficient evidence to proceed with PCA [31]. This value was instrumental in the deciding which variables to select in constructing the wealth index. The combination of socioeconomic variables that produced the greatest KMO value was selected for PCA.

Bartlett’s test of sphericity was used to test the null hypothesis that the variables in the population correlation matrix are uncorrelated. Since the p-value (0.000) for the Bartlett’s statistic was less than 5%, the null hypothesis that the variables in the population correlation matrix are uncorrelated was rejected [31]. It was therefore concluded that there was enough evidence of a significant relationship between the variables used to proceed with PCA.

**Fig 1:**
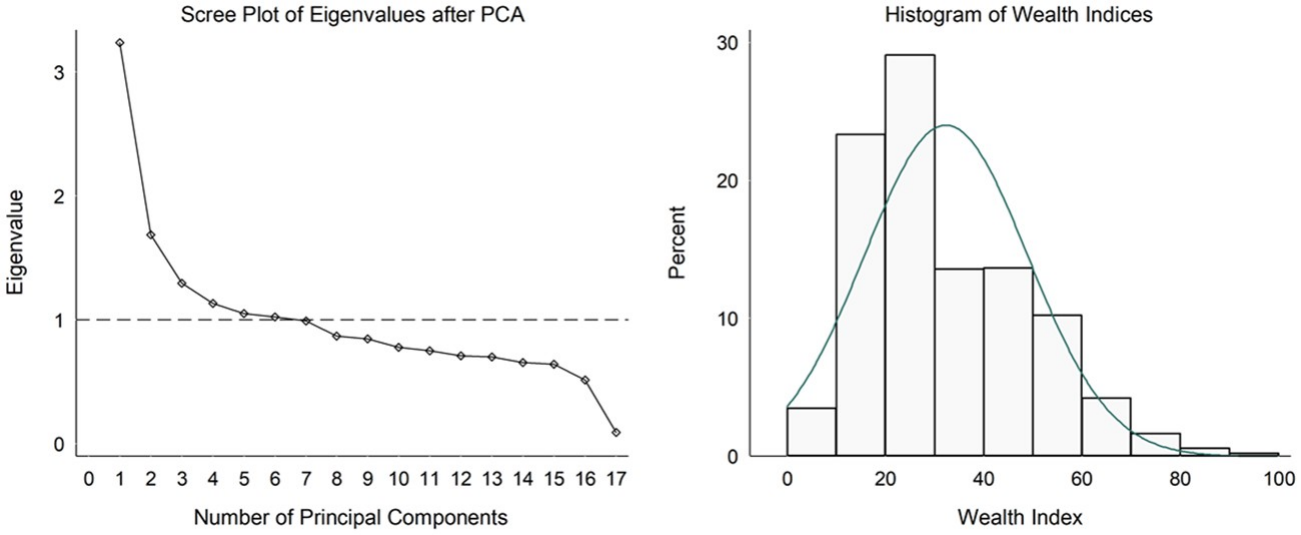
Scree test and histogram of wealth indices derived from PCA.

The scree plot in Fig 1 shows that the first six principal components had eigenvalues that were greater or equal to one, and as a result, these were used to compute the socioeconomic wealth index.

#### Computation of wealth index

To compute the socioeconomic wealth index, linear regression was employed to calculate the principal component scores for each elderly person using the loadings of the first six principal components [31, 32]. The formula used to compute the principal component scores is:

S[i,j]=V[i,j]×PCA[i,j],

where *S*[*i*, *j*] represents the principal component score for elderly *i*; *V*[*i*, *j*] is the value of variable *j* for elderly *i*; and *PCA*[*i*, *j*] represents the principal component loading for elderly *i* (*i* = 1 to 1,830 and *j* = 1 to 17).

The non-standard socioeconomic wealth index (NSI) for each elderly person was computed using the following formula [31]:

$$NSI=\sum_{i=1}^{6}\frac{PROP_{i}}{56}\times PCA_{i\;},$$

where *PROP*_*i*_ is the contribution to the total variation for each of the six PCAs, and *PCA*_*i*_ is the value for principal component *i*

The socioeconomic wealth index was standardized using the formula:

$$Standardized\;wealth\;index=\frac{\left(NSI\;for\;each\;elderly-Min\;NSI\right)}{\left(Max\;NSI-\;Min\;NSI\right)}$$

The above formula transformed the NSI socioeconomic wealth indices into standardized values that range from 0 to 100 [32]. The socioeconomic wealth indices were skewed to the right, which is indicative of income variables (Fig 1) and were categorized into quintiles.

#### Logistic regression model

Two multivariate logistic regression models were employed to examine the relationship between socioeconomic factors and multimorbidity. The logit model examined the effect of socioeconomic characteristics on multimorbidity among the elderly population aged 70 years and over. A binary dependent variable was constructed and defined as:

$$y_{i}=\{\begin{array}{c} 1\;\text{if}\;\text{the}\;\text{elderly}\;\text{person}\;\text{has}\;\text{more}\;\text{than}\;\text{one}\;\text{chronic}\;\text{medical}\;\text{condition}\;\\ \;0\;\text{otherwise}\; \end{array}$$

The standard logit model used to estimate the coefficients of the *β* vector is

$$P(Y_{i}={1|X}_{j})=F(X_{j}\beta )$$

where *Y*_*i*_ = 1 represents the event that the elderly person suffers from at least two medical conditions and *Y*_*i*_ = 0 represents the condition that the elderly does not suffer from more than one medical condition [33]. The variable *X*_*j*_ was further defined as the vector of explanatory variables gender, ethnicity, physical activity, smoking history, educational attainment, and municipality. The function *F*(*z*) is the standard cumulative logit.

An unweighted logit model and a weighted logit model were used to examine the relationship between multimorbidity and selected socioeconomic characteristics of the elderly population. Both models in Table 5 examine the effect of social factors, lifestyle habits, economic status, and geographic characteristics on multimorbidity among the elderly, which are defined by the following variables: gender, age, ethnicity, levels of physical activity among elderly population, smoking history, level of education, wealth index (proxy for income), and municipality. The coefficient estimates reported in Table 5 are the odds ratios for multimorbidity for each of the socioeconomic variables. All analyses were conducted using Stata 14.2.

## Results

### Demographic and socioeconomic characteristics

Of the 1,898 participants in the dementia survey, adequate data for development of the socioeconomic wealth index using PCA, was available for 1,830. The estimates for multimorbidity prevalence and the odds ratio were based on a sample of 1,806 participants in concordance with the data provided.

Socio-economic characteristics of the elderly participants are shown in Table 2. An estimated 56% of the 1,806 elderly respondents were female, while 44% were male. The average age was 78 years. Persons of African and East Indian descent made up the bulk of the elderly respondents, 37% and 38%, respectively. Less than a third of the elderly respondents attained secondary or tertiary education. The respondents were either very much physically active (32%) or fairly physically active (39%). A mere 6% of the 1,806 respondents were not physically active at all. Five percent (5%) of the elderly respondents used to smoke heavily in the past (smoked at least 20 cigarettes a day). Elderly respondents were evenly spread across the five economic quintiles, with 20%, 22%, 18%, 20%, and 20% belonging to quintiles 1, 2, 3, 4 and 5, respectively. Most of the elderly respondents reside in the municipalities of Tunapuna/Piarco (16%), Couva/Tabaquite/Talparo (13%), San Juan/Laventille (12%), and Siparia and Point Fortin (11.2%).

### Morbidity among the elderly

Of the eighteen chronic medical conditions (Table 1) that were used to identify and estimate multimorbidity among the elderly, the most common chronic medical conditions reported were hypertension (45.2%), followed by diabetes (32.2%) and arthritis (29.5%). Prevalence for the remaining 15 chronic medical conditions is presented along with the p-values for the difference between elderly men and women.

**Table 1:** Prevalence of chronic medical conditions used to define multimorbidity.

Chronic disease conditions	Total	Men	Women	p-value
	(%)	(%)	(%)	
Hypertension	45.2	36.6	51.9	<0.0001
Diabetes	32.2	29.5	34.4	0.026
Arthritis	29.5	20.3	36.7	<0.0001
High Cholesterol	13.0	9.1	16.1	<0.0001
Heart Disease	10.7	9.8	11.5	0.264
Stroke	5.4	6.8	4.3	0.022
Cancer	2.4	3.1	1.8	0.058
Asthma	2.3	2.3	2.4	0.884
Alzheimer’s Disease	2.0	1.1	2.8	0.015
Depression	1.9	1.1	2.6	0.028
Parkinson’s Disease	1.4	2.1	0.9	0.027
Dementia	1.3	0.8	1.7	0.082
Prostate Cancer	1.3	2.9	0.0	<0.0001
Angina	1.1	0.9	1.3	0.417
Kidney Disease	0.7	1.0	0.4	0.112
Epilepsy	0.5	0.6	0.4	0.482
Thyroid	0.4	0.1	0.6	0.113
Osteoporosis	0.3	0.0	0.6	0.030

Generally, morbidity among the elderly population in Trinidad and Tobago was more prevalent among elderly females than elderly males. More than half of elderly females reported having been diagnosed with hypertension (51.9%), compared to the elderly male participants (36.6%). Differences in prevalence between elderly men and women were also observed for diabetes (Men 29.5%; Women 34.4%) and arthritis (Men 20.3%, Women 36.7%).

### Prevalence and characteristics of multimorbidity among the elderly

**Fig 2:**
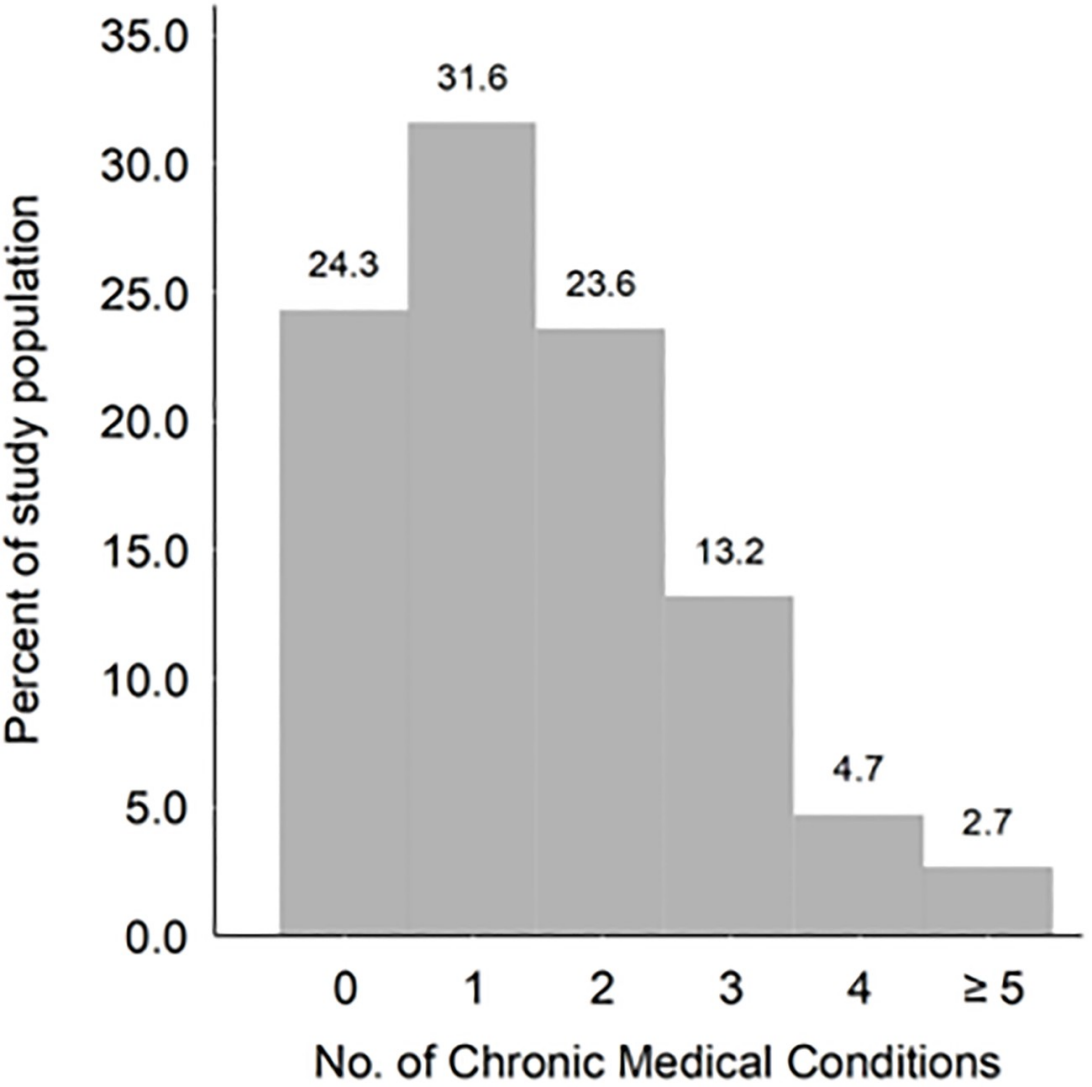
Distribution of the number of chronic medical conditions in study population.

The national prevalence of multimorbidity among the elderly population 70+ was estimated at 44%. Furthermore, 24% of the elderly population was not diagnosed with a chronic medical condition (Fig 2), while approximately 32% were diagnosed with only one chronic medical condition.

Most of the elderly participants (76%) had at least one chronic medical condition. Table 3 shows the unweighted prevalence of multimorbidity among the elderly participants for selected demographic and social variables.

The prevalence of multimorbidity among elderly females was significantly higher than for elderly males, 51% and 35%, respectively (Table 2). The greatest concentration of multimorbidity was among the 70–79 age group, where 65% reported being diagnosed with two or more chronic medical conditions. Six percent (6%) of the multimorbid participants came from the 90+ age group.

**Table 2:** Socioeconomic and demographic characteristics of the elderly with multimorbidity (n = 1,806).

Characteristics	Category	Respondents		Distribution of Multimorbidity		Number with and Prevalence of Multimorbidity	
		Number	%	With (%)	Without (%)	Number	%
*Gender*	Male	794	44.0	34.9	51.1	278	35.0
	Female	1,012	56.0	65.1	48.9	519	51.3
			**100.0**	**100.0**	**100.0**		
*Age Group*	70–74	684	37.9	38.3	37.6	305	44.6
	75–79	493	27.3	28.0	26.8	223	45.2
	80–84	338	18.7	18.2	19.1	145	42.9
	85–89	186	10.3	9.9	10.6	79	42.5
	90+	105	5.8	5.6	5.9	45	42.9
			**100.0**	**100.0**	**100.0**		
*Ethnicity*	African	667	36.9	32.5	40.4	259	38.8
	East Indian	689	38.2	46.3	31.7	369	53.6
	Mixed African & East Indian	90	5.0	3.1	6.4	25	27.8
	Other	360	19.9	18.1	21.4	144	40.0
			**100.0**	**100.0**	**100.0**		
*Physically active*	Very much	576	31.9	19.3	41.8	154	26.7
	Fairly	706	39.1	42.7	36.3	340	48.2
	Not very much	408	22.6	29.5	17.1	235	57.6
	Not at all	116	6.4	8.5	4.8	68	58.6
			**100.0**	**100.0**	**100.0**		
*Smoking History*	Light Smoker	1,709	94.6	94.5	94.7	753	44.1
	Heavy Smoker	97	5.4	5.5	5.3	44	45.4
*Education Attainment*			**100.0**	**100.0**	**100.0**		
	Low Education	1,348	74.6	80.1	70.4	638	47.3
	High Education	422	23.4	17.9	27.7	143	33.9
	Other	36	2.0	2.0	2.0	16	44.4
			**100.0**	**100.0**	**100.0**		
*Wealth Group (Elderly)*	Poorest	359	19.9	17.3	21.9	138	38.4
	2nd	404	22.4	24.3	20.8	194	48.0
	3rd	328	18.2	21.1	15.9	168	51.2
	4th	361	20.0	18.2	21.4	145	40.2
	Richest	354	19.6	19.1	20.0	152	42.9
			**100.0**	**100.0**	**100.0**		
*Municipality*	Port of Spain	34	1.9	1.0	2.6	8	23.5
	Mayaro	39	2.2	3.0	1.5	24	61.5
	Sangre Grande	161	8.9	9.8	8.2	78	48.4
	Princes Town	142	7.9	8.3	7.5	66	46.5
	Penal/Debe	135	7.5	7.4	7.5	59	43.7
	Siparia and Point Fortin	202	11.2	13.4	9.4	107	53.0
	City of San Fernando	72	4.0	3.9	4.1	31	43.1
	Borough of Arima	51	2.8	2.3	3.3	18	35.3
	Borough of Chaguanas	92	5.1	6.6	3.9	53	57.6
	Diego Martin	144	8.0	4.6	10.6	37	25.7
	San Juan/Laventille	215	11.9	11.5	12.2	92	42.8
	Tunapuna/Piarco	282	15.6	12.3	18.2	98	34.8
	Couva/Tabaquite/Talparo	237	13.1	15.8	11.0	126	53.2
			**100.0**	**100.0**	**100.0**		

Persons of East Indian descent accounted for the majority of the multimorbid participants (46%), while those of African descent constituted 33%. A further 3% were of mixed African and East Indian descent and the remaining 18% constituted other ethnic groups. An examination of multimorbidity within the different ethnic groups, reveals that over 50% of the elderly of East Indian descent reported being diagnosed with two or more chronic medical conditions (54%), compared to 39% for the elderly population of African descent. Multimorbidity prevalence for Mixed African and East Indian and Other ethnic groups was 28% and 40%, respectively.

The prevalence of multimorbidity for elderly persons who were not much physically active (58%) was much higher than that of elderly persons who were very much physically active (27%). In terms of geographic location, prevalence of multimorbidity was above 50% for elderly persons living in the municipalities of Mayaro, Siparia and Point Fortin, Chaguanas, and Couva/Tabaquite/Talparo, all of which are located in the south, east and central of the island of Trinidad.

### Empirical results of PCA

Table 3 presents the percentage of the 1,830 participants who are owners of the 17 socioeconomic variables that were selected for PCA. It also shows the loadings for the first six principal components, which are derived from applying the varimax rotation. The first 6 principal components account for 56% of the total variation. The greatest variations are explained by the first two principal components, with the first principal component accounting for 19.1% of the total variation followed by 9.9% for the second component.

**Table 3:** Summary statistics and first six principal components of 17 dichotomous assets variables.

Socioeconomic Characteristics	PCA 1	PCA 2	PCA 3	PCA 4	PCA 5	PCA 6	Means	SD
*Physical Assets*								
Microwave Oven	.	.	.	0.4142	.	.	0.79	0.41
Deep Freezer	.	.	.	.	.	.	0.33	0.47
DVD Player	.	.	.	0.5496	.	.	0.47	0.50
Basic TV	-0.4409	.	.	.	.	.	0.28	0.45
Mobile Phone	.	.	.	0.6198	.	.	0.77	0.42
*Financial Assets*								
Unit Trust or Shares	.	.	0.4911	.	.	.	0.07	0.26
Retirement Annuity	.	.	0.5494	.	.	.	0.02	0.15
Life Insurance	.	.	0.4660	.	.	.	0.04	0.20
Funeral Policy	.	.	0.4561	.	.	.	0.01	0.12
*Computer*								
Personal Computer in Household	0.5964	.	.	.	.	.	0.33	0.47
Household has Access to Internet	0.6011	.	.	.	.	.	0.29	0.45
*Educational Attainment*								
Primary	.	-0.5351	.	.	.	.	0.70	0.46
Tertiary	.	0.5487	.	.	.	.	0.07	0.25
*Occupation (Current or Former)*								
Professional	.	0.5572	.	.	.	.	0.11	0.31
Semi-skilled Labourer	.	.	.	.	0.7452	.	0.08	0.27
Unskilled Labourer	.	.	.	.	-0.6456	.	0.15	0.36
*Health Programmes*								
Chronic Disease Assistance Programme	.	.	.	.	.	0.8749	0.71	0.45
Eigenvalues for PCAs	3.240	1.689	1.299	1.134	1.052	1.024		
Proportions for PCAs	0.191	0.099	0.076	0.067	0.062	0.060		
Cumulative proportions for PCAs	0.191	0.290	0.366	0.433	0.495	0.555		

The first principal component represents the assets associated with the computer (internet access and possession of a computer). Having basic TV seems to reduce the value for this principal component. Principal component 2 represents the educational qualifications of the elderly. Principal component 3 represents the financial assets of the elderly person’s household, while principal component 4 represents ownership of personal (mobile phone) and household assets items (microwave oven, deep freezer, DVD player). The elderly participants who used to work in semi-skilled and unskilled occupations are represented using principal component 5, and users of the CDAP drugs are represented by principal component 6.

### Results of wealth index—Economic variable

Table 4 reports the quintile distribution of the 17 socioeconomic variables selected to compute the wealth index. Missing values for the selected socioeconomic variables were imputed.

**Table 4:** Distribution of socioeconomic assets by quintile from PCA.

Socioeconomic Characteristics	Quintile 1	Quintile 2	Quintile 3	Quintile 4	Quintile 5	All Quintiles
Microwave Oven	0.38	0.74	0.92	0.94	0.98	0.79
Deep Dreezer	0.08	0.14	0.41	0.37	0.67	0.33
DVD Player	0.18	0.30	0.54	0.62	0.76	0.47
Basic TV	0.64	0.33	0.15	0.12	0.05	0.28
Mobile Phone	0.50	0.79	0.76	0.85	0.92	0.77
Unit Trust or Shares	0.00	0.01	0.02	0.07	0.22	0.07
Retirement Annuity	0.00	0.00	0.00	0.01	0.08	0.02
Life Insurance	0.00	0.00	0.00	0.07	0.16	0.04
Funeral Policy	0.00	0.00	0.00	0.01	0.06	0.01
Personal Computer in Household	0.01	0.01	0.10	0.61	0.96	0.33
Household has Access to Internet	0.00	0.00	0.01	0.51	0.94	0.29
Primary Education	0.90	0.82	0.71	0.64	0.35	0.70
Tertiary Education	0.00	0.00	0.02	0.09	0.24	0.07
Professional	0.00	0.02	0.07	0.16	0.31	0.11
Semi-skilled Labourer	0.01	0.08	0.14	0.10	0.08	0.08
Unskilled Labourer	0.36	0.21	0.07	0.14	0.02	0.15
Chronic Disease Assistance Programme	0.51	0.77	0.80	0.67	0.77	0.71
Count	368	407	332	365	358	1830
Means of wealth index	13.20	21.78	28.89	40.55	58.64	32.30
Standard Deviations of wealth index	3.85	2.21	2.09	4.45	9.63	16.62

### Empirical results of the logistic regression model

Both models demonstrate that the odds ratios for age, gender, and ethnicity are statistically significant, and suggest that elderly women are two times more prone to multimorbidity than elderly men. The odds ratios for gender are significant at the 1% level. Although the odds ratios are significant for age, the direction of the influence is somewhat unexpected. Age was used as a continuous predictor variable in both models. Accordingly, the odds ratios for age correspond to one-year intervals. For the elderly person who is 70+, the results in Table 5 show that the odds of multimorbidity increase by 0.97 for a one year increase in age. This finding suggests that the odds of multimorbidity do not increase with age.

**Table 5:** Multivariate logistic analysis of association between socioeconomic characteristics and multimorbidity in the elderly population of Trinidad and Tobago.

Characteristics	Unweighted Model			Weighted Model		
	Odds ratio	95% CI	P-value	Odds ratio	95% CI	P-value
***Gender***						
Male	1.00			1.00		
Female	1.94***	[1.57,2.40]	0.00	2.07***	[1.67,2.58]	0.00
***Age***						
Age of elderly	0.97***	[0.96,0.99]	0.00	0.97***	[0.95,0.99]	0.01
***Ethnicity***						
African	1.00			1.00		
East Indian	1.42***	[1.11,1.82]	0.01	1.42**	[1.05,1.91]	0.02
Mixed African and East Indian	0.58**	[0.35,0.99]	0.04	0.59*	[0.34,1.02]	0.06
Other	1.06	[0.80,1.41]	0.69	1.04	[0.78,1.39]	0.78
***Level of physical activity***						
Very much	1.00			1.00		
Fairly	2.46***	[1.91,3.18]	0.00	2.38***	[1.81,3.14]	0.00
Not very much	3.62***	[2.68,4.88]	0.00	3.55***	[2.64,4.76]	0.00
Not at all	4.03***	[2.55,6.37]	0.00	3.82***	[2.62,5.59]	0.00
***Smoking History***						
Light Smoker	1.00			1.00		
Heavy Smoker	1.60**	[1.01,2.53]	0.04	1.64*	[0.98,2.76]	0.06
***Educational Attainment***						
High Education	1.00			1.00		
Low Education	1.33**	[1.01,1.76]	0.04	1.40**	[1.04,1.88]	0.03
Other	1.06	[0.51,2.20]	0.88	1.19	[0.58,2.41]	0.63
***Economic Status***						
Poorest	1.00			1.00		
2nd	1.41**	[1.03,1.92]	0.03	1.51**	[1.10,2.08]	0.01
3rd	1.88***	[1.35,2.61]	0.00	1.99***	[1.38,2.87]	0.00
4th	1.32	[0.95,1.84]	0.10	1.44**	[1.01,2.05]	0.05
Richest	1.88***	[1.31,2.69]	0.00	1.95***	[1.30,2.94]	0.00
***Municipality***						
Port of Spain	1.00			1.00		
Mayaro	2.68*	[0.90,7.94]	0.08	2.81	[0.76,10.39]	0.12
Sangre Grande	2.06	[0.84,5.07]	0.12	2.30	[0.65,8.18]	0.19
Princes Town	2.14*	[0.87,5.27]	0.10	2.20	[0.62,7.87]	0.22
Penal/Debe	1.64	[0.66,4.09]	0.29	1.74	[0.45,6.69]	0.42
Siparia and Point Fortin	2.13*	[0.88,5.18]	0.10	2.35	[0.66,8.34]	0.18
City of San Fernando	1.68	[0.64,4.44]	0.29	1.85	[0.44,7.83]	0.40
Borough of Arima	1.45	[0.52,4.08]	0.48	1.59	[0.39,6.49]	0.52
Borough of Chaguanas	2.60**	[1.01,6.66]	0.05	2.67	[0.75,9.54]	0.13
Diego Martin	0.92	[0.37,2.31]	0.86	1.00	[0.27,3.73]	0.99
San Juan/Laventille	1.73	[0.72,4.14]	0.22	1.87	[0.51,6.81]	0.34
Tunapuna/Piarco	1.21	[0.50,2.89]	0.67	1.33	[0.37,4.83]	0.66
Couva/Tabaquite/Talparo	2.34*	[0.97,5.64]	0.06	2.53	[0.70,9.06]	0.15
Observations	1806			1806		

Odds ratios; 95% C.I in parentheses.

* *p* < 0.1,

** *p* < 0.05,

*** *p* < 0.01.

The odds ratios from both logistic models show at the 5% level that an elderly person of East Indian descent is at least 1.4 times more prone to multimorbidity than an elderly person of African descent.

## Discussion

Consistent with results of a study undertaken by Rawlins et al. [34] on the health of the elderly in Trinidad, hypertension, arthritis, and diabetes were found to be the top three most reported chronic medical conditions among the elderly. Further, the prevalence of hypertension and diabetes for both men and women in this study was also similar to Rawlins et al’s findings.

Interestingly, Rawlins et al. [34] reported that 80% of the elderly sampled reported at least one chronic disease, with 37% reporting one chronic disease, 23% reporting two chronic diseases and 20% reporting 3 or more chronic diseases. This study reports that 76% of the participants have at least one chronic medical condition, with 33% having only one chronic medical condition, 23% with two and 20% with 3 or more chronic medical conditions. Rawlins et al. [34] based their findings on 5 specific chronic disease conditions (hypertension, diabetes, arthritis, heart disease, and stroke), and an unspecified chronic disease condition, which was identified as chronic medical problems.

The national estimate of multimorbidity reported in this study is lower than some estimates found in the literature. Salive [20] reported that multimorbidity studies that focused on the elderly population generally produced estimates of multimorbidity that were greater than 50%. Although the study by Rawlins et al. did not focus specifically on multimorbidity, there was sufficient data to obtain an estimate for the prevalence of multimorbidity among the elderly in Trinidad of 43%, which is comparable to our estimate of 44%. Prevalence of multimorbidity of less than 50% among the elderly has been reported in the literature as per Jerliu et al. [15], who reported prevalence of multimorbidity of 45% for persons aged 65+ in Kosovo.

The findings from the logistic regression analysis corroborate previous studies suggesting that multimorbidity is more frequent among female [13], increases with age [18], associated with low education [5, 24], linked to smoking history [11] and related to lack of physical activity [35].

The higher prevalence of multimorbidity among elderly women may be explained by the fact that women generally use the health services more frequently than men [36]. Even though age was a significant predictor in this study, the magnitude of its effect on multimorbidity was less than one [OR = 0.97]. The exclusion of data on persons in the age range 60–69 in the logit models could have accounted for the low odds ratios generated by the models.

As indicated in the results, an elderly person of East Indian descent is at least 1.4 times more likely to have multimorbidity than an elderly person of African descent. Studies have shown that the prevalence of diabetes, hypertension, and heart disease is higher among persons of East Indian descent [37] than persons of African descent.

Hypertension, diabetes and heart disease in Trinidad and Tobago are more prevalent among persons with lower education [37]. This study provides additional evidence that education plays a significant role in reducing multimorbidity among the elderly in Trinidad and Tobago. Elderly persons with lower than secondary level education are more prone to multimorbidity than persons reporting a higher education level.

Although some studies report that multimorbidity increases with lower income [38], the results of the two logistic regression models show a reverse effect of income on multimorbidity. The odds ratios reported in Table 5 seem to suggest that elderly persons in the upper quintiles are generally more prone to multimorbidity in Trinidad and Tobago. Alaba and Chola (2013) reported similar findings using data from the South Africa National Income Dynamics Study (SA-NIDS) [11]. This is not the first study on the Caribbean to report that the poor experience better levels of health than the non-poor. According to Brown (2006), the very character of poverty—lower levels of education, training and literacy, social exclusion and marginalization—work together to foster a lack of awareness of illness that may not be self-evident or obvious among the poor [39]. This, he argues, leads to the poor being less likely to perceive the symptoms of disease than the more privileged non-poor, thereby contributing to underreporting of illness by the poor relative to the non-poor. The findings of our study are also consistent with that of La Foucade and Scott (2006) with respect to patterns of reported ill health in the Caribbean [40].

These results contrast with those of Khanam et al. [3], who reported that multimorbidity was significantly higher among the poorest members of the population in Bangladesh, and Hosseinpoor et al. [38], who highlighted higher multimorbidity among lower quintile populations and several middle and lower income countries. It may be that information bias due to underreporting by the poor contributed to the contradiction between these results and those of this study.

This study used a proxy variable (using PCA) for income, due to the non-response rate for the income variable of 71% for the dementia survey. The proxy variable was, for the most part, consistent with our expectations—an elderly person in the upper quintile households is more likely to hold financial assets such as unit trust shares, retirement annuities, life insurance and funeral policies, than an elderly person in the lower quintile (Table 5). The proxy variable shows that an elderly person from the upper quintiles is more likely to have worked as a professional in the past, more likely to have attained tertiary education compared to his/her counterpart in the lower quintiles. One possible reason for the higher multimorbidity among the wealthier elderly in this study is the general tendency of wealthier persons to utilize the services of a physician more than those in the lower quintiles, thus leading to definitive diagnoses of chronic diseases [41]. Further, Trinidad and Tobago also has high out-of-pocket payments (OOP) for health care services (44% of total health expenditure) [42], which can lead to decreased utilization of health services particularly among lower population quintiles [43].

### Strengths and limitations

A major strength of this study is that the data was derived from a representative sample of the elderly population in Trinidad and Tobago, which allows for generalization of the findings to the entire population of elderly persons 70 and over. This study was able to provide population-based estimates of multimorbidity among the elderly, along with identifying the associated socioeconomic characteristics. The 18 chronic conditions included in this study incorporated the top chronic conditions (hypertension, diabetes, and arthritis) affecting the elderly population in Trinidad and Tobago.

One limitation of this study is the exclusion of the elderly population on the island of Tobago in the dementia survey. Since, the population of Tobago accounts for only 5% of the total population of Trinidad and Tobago, multimorbidity prevalence and the socioeconomic factors associated with it on both islands would have to vary significantly to affect the estimates presented in this study.

Multimorbidity was measured by a simple count of self-reported chronic medical conditions. Using this measurement approach means that altering the number of medical conditions could have altered the prevalence of multimorbidity and the multivariate logistic results in this study. Additionally, there is the possibility that participants who reported having one or none of the listed morbidities could have other unlisted chronic medical conditions since the medical conditions were self-reported and not measured independently by a health professional. This could have led to some degree of underreporting.

A further limitation of this study is the absence of alternative multimorbidity indices, such as the Index of Coexisting Diseases or the Cumulative Illness Rating Scale [44], that measure the duration and severity of the disease or medical condition.

Finally, the possibility of reverse causality cannot be ignored. The findings of the logistic regression analysis show that the burden of multimorbidity is strongly associated with little or no physical activity. It is entirely possible that little or low physical activity could be the result of the multimorbid condition of the elderly person [45].

## Conclusion

In this study, we described the epidemiology of multimorbidity among the elderly population of Trinidad and Tobago and investigated the relationship between multimorbidity and socioeconomic factors in that population. Using a nationally represented sample of elderly persons 70 years and older, it was demonstrated that multimorbidity among the elderly is strongly associated with social, lifestyle, and economic factors. Social and demographic factors such as gender, ethnicity, and education were found to significantly impact multimorbidity. Lifestyle factors such as the level of physical activity and smoking history were found to increase the probability of multimorbidity among the cohort. The impact of economic factors on multimorbidity, measured by developing a socioeconomic wealth index, using PCA, were somewhat unexpected. The evidence shows that diagnosis of multiple medical conditions is more prevalent among elderly persons in the higher socioeconomic group than elderly persons in the lowest socioeconomic group.

A better understanding of the socioeconomic characteristics associated with multimorbidity can help identify the subdivisions of the elderly population that are at the greatest risk of multimorbidity. The findings of the study can be useful in developing guidelines and policies to reduce multimorbidity among the elderly population in Trinidad and Tobago. Any such policies should encourage greater levels of physical activity, provide education on the risk factors of multimorbidity, and discourage smoking. This can be achieved by linking primary and preventive care during the life cycle to explicitly defined screening for major health risks, more so in the 50 years and above cohort. Coverage of the cost of these screenings by the State may incentivize persons to access them and may prove to be an early warning system of sorts. This, together with the current CDAP initiative, which provides medication for chronic diseases free of charge to recipients, can yield positive results in identifying, treating, and reducing morbidity in the elderly.

## Supporting information

S1 File (PDF)

S2 File (DTA)

S3 File (DTA)
