# Supplementary material for: The socio-economic determinants of multimorbidity among the elderly population in Trinidad and Tobago
Source: PLoS One. 2020 Sep 11;15(9):e0237307. doi: 10.1371/journal.pone.0237307 (PMC7485802; doi:10.1371/journal.pone.0237307)
Supplement: S1 File — (PDF) [file pone.0237307.s001.pdf]

Administrative Code

THE UNIVERSITY OF THE WEST INDIES
ST AUGUSTINE, TRINIDAD AND TOBAGO, WEST INDIES

The Prevalence and Economic Cost of Dementia in Trinidad & Tobago Project

2014

A collaboration between
The Faculty of Medical Sciences
&
HEU, Centre for Health Economics,
Faculty of Social Sciences

Confidential

47614

INSTRUCTIONS FOR FILLING OUT QUESTIONNAIRE

1) Use only black ink ball point pen.
2) Place an "X" in the box for choice options.
3) Please print in capital letters and avoid contact with the edge of the box.

Correct mark ☒

785

Municipality I.D.	E.D. Number	Building Number	Dwelling Unit Number	Household Number

Respondent's Name: ______________________________

Address of Household:

No. Street Name

Community

Region

Email:

Municipality/Region/ Parish: ______________________

Telephone Number: ___ - ____

Number of persons in household:

VISIT	DATE DD	MM	YYYY	TIME ARRIVED	TIME LEFT	RESULT CODE
1				:	:	☐ 1 ☐ 2 ☐ 3 ☐ 4 ☐ 5 ☐ 6 ☐ 7
2				:	:	☐ 1 ☐ 2 ☐ 3 ☐ 4 ☐ 5 ☐ 6 ☐ 7
3				:	:	☐ 1 ☐ 2 ☐ 3 ☐ 4 ☐ 5 ☐ 6 ☐ 7
4				:	:	☐ 1 ☐ 2 ☐ 3 ☐ 4 ☐ 5 ☐ 6 ☐ 7

Result Code:
- 1 Completed
- 2 Partially completed
- 3 Not at home
- 4 Refused
- 5 Vacant dwelling
- 6 Closed dwelling
- 7 Other: ______________________ (SPECIFY)

Interviewer's Code: Signature: ______________________ Date: dd / mm / yyyy

Supervisor's Code: Signature: ______________________ Date: dd / mm / yyyy

47614

INTRODUCTION AND CONSENT

YOU ARE REQUIRED TO READ THE FOLLOWING TO THE HEAD OF THE HOUSEHOLD

Hello,

My name is _________________________ and I am conducting research for Faculty of Medical Sciences and Social Sciences, UWI. The study is "The Prevalence and Economic Cost of Dementia in Trinidad and Tobago". The information collected from this survey could help improve the health care system in Trinidad and Tobago, in particular, as it relates to the care and support for persons over 60 years and their families.

The Questions will be addressed to three (3) persons (respondents):

1. The Head of the Household;
2. The Participant (elderly person selected for the study); and
3. A Proxy (relative, friend etc.).

Consent will be obtained from the head of household and the participant to administer the relevant sections of the Questionnaire Booklet. This entire process which has three parts, should take less than one hour.

OBTAINING CONSENT FROM THE RESPONDENTS

HEAD OF HOUSEHOLD

I want to ask you questions about your household for example, your household's composition, economic activity, etc. Your participation is entirely voluntary. You can choose not to participate if you do not want to. Also, you can refuse to answer any question and you can stop the interview at any time. All information collected will be kept confidential. If you have any questions about this study at any time you can contact Mrs. Sherma Robinson at 683 7604.

After reading the statement above, you should ask the Head of Household:

HEAD OF HOUSEHOLD

a. Do you have any questions right now? ☐ 1. Yes ☐ 2. No

b. Do you wish to proceed with this interview? ☐ 1. Yes ☐ 2. No

PARTICIPANT (Elderly person selected for the study)

I want to ask you questions that relate to your memory and concentration. We are interested in how these affect people's health and well-being. Your participation is entirely voluntary. You can choose not to participate if you do not want to. Also, I will like to talk to your (relative, friend etc.) asking him/her questions about you. All information collected will be kept confidential. If you have any questions about this study at any time you can contact Mrs. Sherma Robinson at 683 7604.

After reading the statement above, you should ask the Participant:

ELDERLY

c. Do you have any questions right now? ☐ 1. Yes ☐ 2. No

d. Do you wish to proceed with this interview? ☐ 1. Yes ☐ 2. No

MARK CHOICE BOXES LIKE THIS ☒

DEMOGRAPHIC DATA SHEET

1.0 AGE, GENDER & EDUCATION

1.1 Sex ☐ 1 Male ☐ 2 Female

1.2 Date of Birth ☐☐ / ☐☐ / ☐☐☐☐ (*dd* / *mm* / *yyyy*)

1.3 Age ☐☐☐

1.4 Age bracket
- ☐ 01. 60-69 years
- ☐ 02. 70-79 years
- ☐ 03. 80-89 years
- ☐ 04. 90+ years

1.5 Highest Level of Education
- ☐ 1. None
- ☐ 2. Primary
- ☐ 3. Secondary
- ☐ 4. Tertiary
- ☐ 5. Other

2.0 ETHNICITY, RELIGION, MARITAL STATUS

2.1 To which ethnic group do you belong?
- ☐ 1. African
- ☐ 2. Caucasian
- ☐ 3. Chinese
- ☐ 4. East Indian
- ☐ 5. Mixed-African & Indian
- ☐ 6. Indigenous
- ☐ 7. Mixed other
- ☐ 8. Portuguese
- ☐ 9. Syrian/Lebanese
- ☐ 10. Other ethnic group
- ☐ 11. Not stated

2.2 To which religion do you belong?
- ☐ 1. Anglican
- ☐ 2. Baptist-Spiritual/Shouter
- ☐ 3. Baptist-other
- ☐ 4. Hindu
- ☐ 5. Islam
- ☐ 6. Jehovah's Witness
- ☐ 7. Methodist
- ☐ 8. Moravian
- ☐ 9. Orisha
- ☐ 10. Pentecostal/Full Gospel/Evangelical
- ☐ 11. Presbyterian
- ☐ 12. Rastafarian
- ☐ 13. Roman Catholic
- ☐ 14. Seventh Day Adventist
- ☐ 15. Atheist
- ☐ 16. None
- ☐ 17. Not stated

2.3 What is your current marital status?
- ☐ 1. Divorced
- ☐ 2. Legally separated
- ☐ 3. Married/co-habiting
- ☐ 4. Never married
- ☐ 5. Widowed
- ☐ 6. Not stated

3.0 OCCUPATIONAL LEVEL

3.1 Are you currently employed? ☐ 1. Yes ☐ 2. No

3.2 What is/was your primary occupation for the majority of your work life?
- ☐ 1. Agricultural worker (self-employed)
- ☐ 2. Agricultural worker (working for others)
- ☐ 3. Associate professional (e.g. technical, nursing, artistic)
- ☐ 4. Clerical worker/secretary
- ☐ 5. Housewife (NB only code if no other job)
- ☐ 6. Manager/Administrator
- ☐ 7. Professional (e.g. health, teaching, legal, financial)
- ☐ 8. Semi-skilled labourer (e.g. helper of skilled labourer)
- ☐ 9. Skilled labourer (e.g. building, electrical etc.)
- ☐ 10. Unskilled labourer

4.0 ACCOMMODATION

4.1 Establish type of accommodation
- ☐ 00. Detached house
- ☐ 01. Condominium
- ☐ 02. Townhouse
- ☐ 03. Geriatric care home
- ☐ 04. Other

4.2 Establish tenure of accommodation
- ☐ 00. Geriatric care home
- ☐ 01. Privately owned by children/family
- ☐ 02. Privately owned by participant/spouse
- ☐ 03. Rented from NHA/housing association
- ☐ 04. Rented from private landlord
- ☐ 05. Warden controlled or 'sheltered' housing
- ☐ 06. Other

47614

5.0 SOCIAL NETWORK

Do you live ...

- ☐ 1. By yourself/alone
- ☐ 2. With spouse
- ☐ 3. With spouse and children
- ☐ 4. With children alone
- ☐ 5. With other (specify) ______________________

6.0 SOCIAL SERVICES

In the last 3 months have you been: (SELECT ALL THAT APPLY)

- ☐ 1. To a general practitioner (private or public)
- ☐ 2. To a hospital (private)
- ☐ 3. To a hospital (public)
- ☐ 4. To a physiotherapist
- ☐ 5. To a traditional healer
- ☐ 6. To a dentist
- ☐ 7. To a specialist

7.0 MEDICAL CONDITIONS

Have you had any of the following conditions in the last 3 months? (SELECT ALL THAT APPLY)

	Yes	No		Yes	No		Yes	No
7.1 Angina	☐	☐	7.7 Depression	☐	☐	7.13 Hypertension	☐	☐
7.2 Alzheimer's disease	☐	☐	7.8 Diabetes	☐	☐	7.14 Parkinson's disease	☐	☐
7.3 Arthritis	☐	☐	7.9 Epilepsy	☐	☐	7.15 Stroke	☐	☐
7.4 Asthma	☐	☐	7.10 Head injury	☐	☐	7.16 Other	☐	☐
7.5 Cancer	☐	☐	7.11 Heart disease	☐	☐	7.17 None	☐	☐
7.6 Dementia	☐	☐	7.12 High cholesterol	☐	☐			

8.0 MEDICATIONS

In the last 3 months, have you taken any of the following tablets or medication? (SELECT ALL THAT APPLY)

- ☐ 1. Medication for dementia
- ☐ 2. Medication for high blood pressure
- ☐ 3. Aspirin
- ☐ 4. Medication to thin blood
- ☐ 5. Medication for diabetes/sugar
- ☐ 6. Insulin
- ☐ 7. Medication for pain
- ☐ 8. Medication for high cholesterol
- ☐ 9. Vitamins
- ☐ 10. Other (specify) ______________________

9.0 IMPAIRMENT LEVEL

Please answer using coding system 0-3 from manual.

Eyesight ☐ Hearing ☐ Climbing stairs ☐ Walking ☐ Ability to speak and understand ☐

10.0 INSTRUMENTAL ACTIVITIES OF DAILY LIVING (IADL)

Are you able to perform the following tasks? (SELECT ALL THAT APPLY)

	Yes	No		Yes	No
10.1 Use the telephone	☐	☐	10.5 Perform laundry	☐	☐
10.2 Shop independently	☐	☐	10.6 Commute independently	☐	☐
10.3 Prepare food independently	☐	☐	10.7 Independently handle finance	☐	☐
10.4 Perform housekeeping	☐	☐	10.8 Take your own medications	☐	☐

11.0 LIFESTYLE: EXERCISE

Taking into account both work and leisure, would you say that you are physically active?

☐ 1. Very much ☐ 2. Fairly ☐ 3. Not very much ☐ 4. Not at all

12.0 LIFESTYLE: SMOKING

**12.1 Have you ever smoked cigarettes/tobacco?**	☐ 1. Yes **(GO TO 12.2)** ☐ 2. No **(SKIP TO 13.1)**
**12.2 How old were you when you started smoking?**	☐☐☐ *(years)*
**12.3 Do you currently smoke?**	☐ 1. Yes **(ANSWER 12.4 & 12.7 ONLY)** ☐ 2. No **(SKIP TO 12.5)**
**12.4 How many cigarettes do you currently smoke daily?**	☐☐☐ *(cigarettes)*
**12.5 How old were you when you stopped smoking?**	☐☐☐ *(years)*
**12.6 How many cigarettes did you smoke daily?**	☐☐☐ *(cigarettes)*
**12.7 Have you ever smoked marijuana regularly?**	☐ 1. Yes ☐ 2. No

13.0 LIFESTYLE: ALCOHOL

13.1 How often do you normally have a drink of something with alcohol in it?

☐ 00. Never ☐ 01. Less than once a month ☐ 02. Less than once a week ☐ 03. At least weekly

13.2 How many alcoholic drinks would you have in an average week? ☐☐

Introduction to Cognitive Section

I am going to ask some questions to do with your memory and concentration. One of the things we are interested in is how these affect people's health and wellbeing.

1. Have you had any difficulty with your memory over the last month? ☐ 1. Yes **(GO TO 1b)** ☐ 2. No **(SKIP TO 2).**

1b. Is that a problem for you? ☐ 1. No problem ☐ 2. Mild problem ☐ 3. Major problem (i.e. significantly concerns them)

2. Have you tended to forget things recently? ☐ 1. Yes **(GO TO 2a and 2b)** ☐ 2. No **(SKIP TO 2c).**

2a/b. What kind of things? Names of family or close friends? What about where you have put things?

2a. Forgets names of family or close friends?

☐ 1. Never or a transient occurrence

☐ 2. Causes difficulty several times per week

☐ 3. Causes difficulty daily

2b. Forgets where they have put things?

☐ 1. Never or a transient occurrence

☐ 2. Causes difficulty several times per week

☐ 3. Causes difficulty daily

2c. Do you have to make more effort to remember things than you used to? ☐ 1. No/Not a problem ☐ 2. Yes

Ok. I'm going to run a few short tests of memory and concentration now. You will find some of these very easy and some will be quite hard. Please bear with me as we need to ask everyone the same questions . . .

Section 1 - COGNITIVE TESTS

Site: ______________________ **Participant ID:** ☐☐☐☐☐☐

I am now going to read out a list of words. Please listen carefully, as I will ask you to repeat them back to me when I have finished.
Read out the 10 words, pausing for one second between each. Mark with an "X" correct words on the grid below.

	1ST	2ND	3RD	
BUTTER	☐	☐	☐	BUTTER
ARM	☐	☐	☐	ARM
LETTER	☐	☐	☐	LETTER
QUEEN	☐	☐	☐	QUEEN
TICKET	☐	☐	☐	TICKET
GRASS	☐	☐	☐	GRASS
CORNER	☐	☐	☐	CORNER
STONE	☐	☐	☐	STONE
BOOK	☐	☐	☐	BOOK
STICK	☐	☐	☐	STICK
**TOTAL SCORE**	☐☐	☐☐	☐☐	

1ST TRIAL
1. Now please tell me all the words you can remember.
Interviewer - Score total number of words correctly recalled in the box above.

2ND TRIAL
Thank you. Now I will read out the words to you one more time. Again, please listen carefully, as I will ask you to repeat the words when I have finished.
Interviewer - Read out the 10 words, pausing for one second between each.

2. Now please tell me all the words you can remember.
Interviewer - Score total number of words correctly recalled in the box above.

3RD TRIAL
Thank you. Now I will read out the words to you one last time. Again, please listen carefully, as I will ask you to repeat the words when I have finished.
Interviewer - Read out the 10 words, pausing for one second between each.

3. Now please tell me all the words you can remember.
Interviewer - Score total number of words correctly recalled in the box.

CSI-D

4. NAME
I'd like you to remember my name.
My last name is xxxxxxx. Can you repeat that please?

☐ 0. Cannot repeat name ☐ 1. Successfully repeats name

We will begin with naming things. I will point to something and I would like you to tell me the name of the object. For example:

	Incorrect	Correct
**5. PENCIL** *(Interviewer shows a pencil)* **What is this called?**	☐ 0	☐ 1
**6. WATCH** *(Interviewer points to his/her watch)* **What is this?**	☐ 0	☐ 1
**7. CHAIR** *(Interviewer pats chair)* **What about this?**	☐ 0	☐ 1
**8. SHOES** *(Interviewer point to shoes [or socks or stockings if they have left shoes outside])* **And these?**	☐ 0	☐ 1
**9. KNUCKLE** *(Interviewer shows his/her knuckles)* **What do we call these?**	☐ 0	☐ 1
**10. ELBOW** *(Interviewer points to his/her elbow)* **What do we call this?**	☐ 0	☐ 1
**11. SHOULDER** *(Interviewer points to his/her shoulder)* **What do we call this?**	☐ 0	☐ 1

I was just showing you things and you told me what we call them. Now I will tell you the name of something and I want you to describe what it is. For example:

	Incorrect	Correct
**12. BRIDGE**<br>**What is a bridge?**<br>**Correct answers: to walk across water, to climb up etc.*	☐ 0	☐ 1
**13. HAMMER**<br>**What do you do with a hammer?**<br>**Correct answers: to drive a nail into something etc.*	☐ 0	☐ 1
**14. PRAY**<br>**What do people do in a** church/temple/mosque (as appropriate)<br>*Correct answers: to pray, to wed.	☐ 0	☐ 1
**15. CHEMIST**<br>**Where do we go to buy medicine?**<br>**Correct answers: chemist, pharmacy etc. (accept locally appropriate answers).*	☐ 0	☐ 1
**16. REPEAT**<br>**Now I would like you to repeat what I say.**<br>*(Only one presentation is allowed, so the* interviewer must read the phrase clearly and slowly, enunciating carefully).<br><br>**'Neither this nor that'**<br>*(exact phrase only)*	☐ 0	☐ 1

17. RECALL
Do you remember that I read out to you a list of words? How many of those words do you remember now? Could you please tell me all the words you can remember.

Interviewer - Score correct words in the grid below.

BUTTER	☐
ARM	☐
LETTER	☐
QUEEN	☐
TICKET	☐
GRASS	☐
CORNER	☐
STONE	☐
BOOK	☐
STICK	☐
**TOTAL SCORE**	☐☐

	Incorrect	Correct
**18. NAME RECALL**<br>Do you remember my name? What is it?	☐ 0	☐ 1

ANIMAL NAMING

Now we are going to do something a little different. I am going to give you a category, and I want you to name, as fast as you can, all of the things that belong in that category. For example, if I say 'articles of clothing' you could say shirt, tie or hat. Can you think of other articles of clothing?

Wait for the subject to give two words. If the subject succeeds, indicate that the responses were correct and proceed to the test itself. If the subject gives an inappropriate word or reply, correct the response and repeat the instructions. If it becomes clear that the subject still does not understand the instructions, terminate this task and explain why this is so. After you are satisfied that the subject understands the task, and has given two words naming articles of clothing, say:

That's fine. I want you to name things that belong to another category, 'animals'. I want you to think about all the many different kinds of animals you know. Think of any kinds of animal in the air, on land, on the water, in the forest, all the different animals. Now I would like for you to tell the names for as many different animals as you can. You will have a minute to do this. Are you ready? Let's begin.

Allow one minute precisely. If the subject stops before the end of the time, encourage him/her to try to find more words. If they are silent for 15 seconds, repeat the basic instruction ('I want you to tell me all the animals you can think of'). No extension on the time limit is made in the event that the instruction has to be repeated.

(The score is the sum of acceptable animals. Any member of the animal kingdom, real or mythical is scored correct, except repetitions and proper nouns. Specifically, each of the following gets credit: a species name and any accompanying breeds within the species; male, female and infant names withing the species).

19. NUMBER OF ANIMALS IN ONE MINUTE ☐☐

Now I am going to tell you three words and I would like you to repeat them after me.
Boat House Fish

Interviewer - Score one point for each correct word on first attempt.

20.1 FIRST TRIAL SCORE

☐ 0. No words remembered ☐ 1. One word remembered
☐ 2. Two words remembered ☐ 3. Three words remembered

Then go on to repeat the three words, up to a total of six times until the subject has remembered them all correctly.

20.2 RECORD NUMBER OF TRIALS UNTIL REPEATED SUCCESSFULLY. ☐☐

Very good. Now try to remember these words because I will be asking you later.

	Incorrect	Correct
**21. TOWN**<br>**What is the name of this city/town/ village (as appropriate)?**	☐ 0	☐ 1
**22. CHIEF**<br>**What is the name of the Prime Minister?**	☐ 0	☐ 1
**23. STREET**<br>**What are the names of the two main streets near here?**<br>*Or (if appropriate)* **What is the name of a river near here?**	☐ 0	☐ 1
**24 . STORE**<br>**Where is the local market/local store?**	☐ 0	☐ 1
**25. ADDRESS**<br>**What is your address?**<br>*Or (if appropriate)* **Who lives next door?**	☐ 0	☐ 1

26. WORD RECALL
Do you remember the three words I told you a few minutes ago?

☐ 0. No words remembered ☐ 1. One word remembered
☐ 2. Two words remembered ☐ 3. Three words remembered

27. LONG TERM MEMORY

	Incorrect	Correct
**Who was the first Prime Minister of Trinidad and Tobago?**	☐ 0	☐ 1

Now I would like to ask some questions about time.

	Incorrect	Correct
**28. MONTH**<br>**What month is it?**	☐ 0	☐ 1
**29. DAY**<br>**What day of the week is it?**	☐ 0	☐ 1
**30. YEAR**<br>**What year is it?**	☐ 0	☐ 1
**31. SEASON**<br>**What season is it?** *(appropriate to the setting).*	☐ 0	☐ 1

I am going to ask you to carry out some actions so please listen carefully, because I will only tell you one time.
(Interviewer - give complete instructions at one time; do not give step by step.)

	Incorrect	Correct
**32. NOD**<br>**Please not your head.**	☐ 0	☐ 1
**33. POINT**<br>**Please point first to the window and then to the door.**	☐ 0	☐ 1

34. PAPER
I'm going to give you a piece of paper. When I do, take the paper in your right hand, fold the paper in half with both hands, and put the paper down on your lap.
Score one point for each component carried out correctly.

Uses right hand ☐ Folds in two ☐ Places in lap ☐

TOTAL SCORE ☐ *(maximum 3)*

35. Now I would like you to take my pencil and copy these figures in the space given below them on the sheet. (See figures on next two sheets).

	Incorrect	Correct
**35.1 CIRCLE**<br>*Score for circles*<br>*Score one if two vaguely circular objects intersect to form a meniscus.*	☐ 0	☐ 1
**35.2 PENTAGONS**<br>*Score for pentagons*<br>*Score one if two five sided objects intersect to form a diamond shape.*	☐ 0	☐ 1

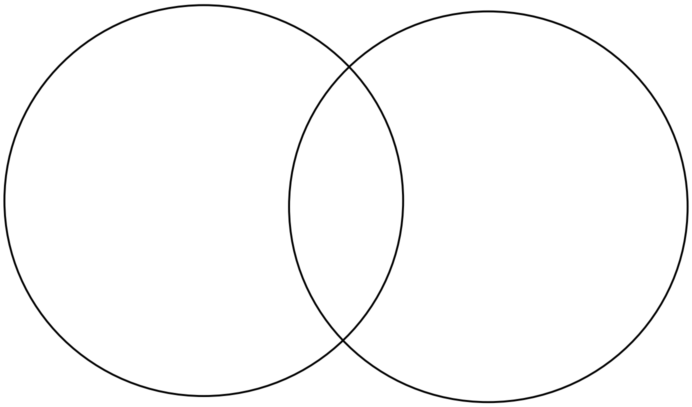

Copy here

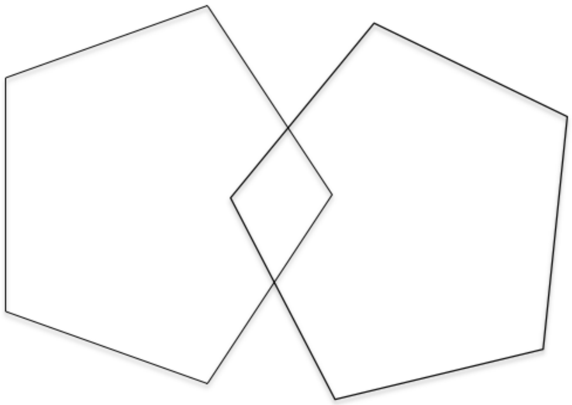

Copy here

Now I will tell a short story, then I will ask you to repeat as much of the story as you can remember. I want you to listen very carefully because I want you to try to tell me the whole story with as many details as you can remember.

Three children were alone at home and the house caught on fire. A brave man managed to climb in a back window and carry them to safety. Aside from minor cuts and bruises, all were well.

Now I would like you to tell me the story in as much detail as possible.

36. STORY
Story recall - total items recalled
Interviewer - score one point for each component correctly recalled.

- ☐ 3 children
- ☐ house on fire
- ☐ brave man climbed
- ☐ children rescued
- ☐ minor injuries
- ☐ everyone well

TOTAL SCORE ☐ *(maximum 6)*

EURO-D SCALE

We are interested in the kind of problems people may have and particularly the way you have been feeling over the past month. It is simply a study of people's possible problems.

Don't be concerned if some questions appear a little odd or strange; some of them will not apply to you, but we have to ask everyone the same sort of questions.

All of the following questions refer to the LAST MONTH.

MH1 [GMS21] DEPRESSION
In the last month, have you been sad or depressed?
QbyQ: If participant asks for clarification, say 'By sad or depressed, we mean miserable, in low spiritis or blue.'

- ☐ 1. Yes
- ☐ 2. No

MH2 [GNS29] PESSIMISM
What are your hopes for the future?

☐ 1. ANY hopes mentioned ☐ 2. NO hopes mentioned

MH3 [GMS30] WISHING DEATH
In the last month, have you felt that you'd rather be dead?

- ☐ 1. Any mention of suicidal feelings or wishing to be dead
- ☐ 2. No such feelings mentioned

QbyQ: This question asks about a specific wish, or preference to be dead. For participants who express ambivalence about living or dying code 2. ANY wish or preference to be dead would be coded 1, even if the participant has felt this only occasionally in the last month.

Examples:
Code 1 Any mention of suicical feelings or wishing to be dead
"Yes"
"Sometimes, I feel that, but not for long"
"I'm looking forward to my death so I can see my husband again"

Code 2 No such feelings mentioned
"I don't really care if I die now, I feel I'm at the end of my life"
"I don't mind. I take each day as it comes"

MH4 [GMS104] GUILT
4.1 Do you tend to blame yourself or feel guilty about anything?

- ☐ 1. Obvious excessive guilt or self-blame **(GO TO MH5)**
- ☐ 2. No such feelings **(GO TO MH5)**
- ☐ 3. Mentions guilt or self-blame, but it is unclear if these constitute obvious or excessive guilt or self-blame **(GO TO MH4.2)**

MH4.2 So for what do you blame yourself?

- ☐ 1. Example(s) given constitute obvious excessive guilt or self-blame
- ☐ 2. Example(s) do not constitute obvious excessive guilt or self-blame, or it remains unclear if these constitute obvious or excessive guilt or self-blame

QbyQ: Only code 1 for an exaggerated feeling of guilt, which is clearly out of proportion to the circumstances. The fault will often have been very minor, if there was one at all. Justifiable or appropriate guilt should be coded 2.

Examples:
Obvious excessive guilt or blame (code 1)
Unusually, very severely depressed people may have lost touch with reality e.g. "The September 11th attack of the World Trade Cenre was my fault. I am to blame."

More commonly, depressed older people feel responsible in an exaggerated way for bringing harm or hurt to those around them. "I am a burden on my children. I am useless and just hold them back."

Trivial, justifiable or appropriate guilt (code 2)
"I left my wife and haven't seen much of my children. I blame myself for this."
"I have not been as kind or considerate to people as I should."

MH5 [GMS54] SLEEP
Have you had trouble sleeping recently?

☐ 1. Trouble with sleep or recent change in sleep pattern

☐ 2. No trouble sleeping

QbyQ - Any trouble sleeping is coded here. Specifically, sleep problems attributed to need to get up to pass water, bodily pain or discomfort, or noisy environment should still be coded 1.

MH6 [GMS113] INTEREST
6.1 In the last month, what is your interest in things?

☐ 1. Less interest than is usual mentioned **(GO TO MH7)**

☐ 2. No change in levels of interest mentioned **(GO TO MH7)**

☐ 3. Non-specific or uncodeable response **(GO TO MH6.2)**

MH6.2 So, do you keep up your interests? ☐ 1. No ☐ 2. Yes

MH7 [GMS105] IRRITABILITY
Have you been more irritable recently? ☐ 1. Yes ☐ 2. No

MH8 [GMS51] APPETITE
8.1 What has your appetite been like?

☐ 1. Diminution in the desire for food **(GO TO MH9)**

☐ 2. No diminution in the desire for food **(GO TO MH9)**

☐ 3. Non-specific or uncodeable response **(GO TO MH8.2)**

QbyQ - It is APPETITE (desire for food) that is being asked about here. Someone who is eating less because of a diet, but does not have any 'diminution in the desire for food' should be coded 1.

MH8.2 So, have you been eating more or less than usual?

☐ 1. Less ☐ 2. More ☐ 3. Neither more nor less

QbyQ - Again, someone who volunteers that they are eating less than usual, but because of a deliberate diet should be coded 3.

MH9 [GMS72] FATIGUE
In the last month, have you had too little energy to do the things you want to do? ☐ 1. Yes ☐ 2. No

MH10 [GMS117/118] CONCENTRATION
10.1 [GMS117] How is your concentration? For example, can you concentrate on a television programme/film/radio?

☐ 1. Difficulty in concentrating on entertainment mentioned

☐ 2. No such difficulty mentioned

QbyQ - Participants who are blind should just be asked about concentrating on a radio programme. Other should be asked about 'a television programme, a film or a radio programme'.

MH10.2 [GMS118] Can you concentrate on something you read?

☐ 1. Difficulty in concentrating on reading mentioned

☐ 2. No such difficulty mentioned

QbyQ - For those who cannot read because of blindness or illiteracy, a missing value code will be entered.

MH11 [GMS114] ENJOYMENT
What have you enjoyed doing recently?

☐ 1. Fails to mention any enjoyable activity

☐ 2. Mentions ANY enjoyment from activity

MH12 [GMS22] TEARFULNESS
In the LAST MONTH have you cried at all? ☐ 1. Yes ☐ 2. No

QbyQ - Any episode of crying would be coded 1 'yes', no matter what the cause. For example, crying over a scene in a film. Do check (if it is unclear from their answer) that the participant is describing crying IN THE LAST MONTH. If necessary, you should repeat the question, stressing this element.

Section 2: INFORMANT QUESTIONNAIRE

Now I am going to ask you some questions about xxxxx. As you know, your xxxxx has given me permission to speak to you about him/her. It is important to have another point of view, as we all find it difficult to describe ourselves.

1.COGNITIVE AND FUNCTIONAL IMPAIRMENT (CSI-D: Section B - Informant Interview)
First of all, I would like to ask a few brief questions about his/her activities nowadays.

I.CSID.1 Have you seen a change in his/her daily activities in the past several years? (if YES, please describe.)

☐ 0. No change

☐ 1. Diminution in range of activities, and/or reduced ability to carry out activities

I.CSID.2 Has there been a general decline in his/her mental functioning?

☐ 0. No change ☐ 1. General decline

I.CSID.3 We all have slight difficulties in remembering things as we get older, but has this been a particular problem for him/her?

☐ 0. No ☐ 1. Yes

Now I would like to ask you other changes you may have noticed in him/her.

I.CSID.4 Does he/she forget where he/she has put things? If YES, how often does that happen?

☐ 0. No ☐ 1. Yes, sometimes ☐ 2. Yes, regularly

I.CSID.5 Does he/she forget where things are usually kept? If YES, how often does that happen?

☐ 0. No ☐ 1. Yes, sometimes ☐ 2. Yes, regularly

I.CSID.6 Does he/she forget the names of friends? If YES, how often does that happen?

☐ 0. No ☐ 1. Yes, sometimes ☐ 2. Yes, regularly

I.CSID.7 Or members of the family? If YES, how often does that happen?

☐ 0. No ☐ 1. Yes, sometimes ☐ 2. Yes, regularly

I.CSID.8 Does he/she forget what he/she wanted to say in the middle of the conversation? If YES, how often does that happen?

☐ 0. No ☐ 1. Yes, sometimes ☐ 2. Yes, regularly

I.CSID.9 When speaking, does he/she have difficulty saying the right words? If YES, how often does that happen?

☐ 0. No ☐ 1. Yes, sometimes ☐ 2. Yes, regularly

I.CSID.10 Does he/she use the wrong words? If YES, how often does that happen?

☐ 0. No ☐ 1. Yes, sometimes ☐ 2. Yes, regularly

I.CSID.11 Does he/she tend to talk about what happened long ago rather than the present? If YES, how often does that happen?

☐ 0. No ☐ 1. Yes, sometimes ☐ 2. Yes, regularly

I.CSID.12 Does he/she forget when he/she last saw you? If YES, how often does that happen?

☐ 0. No ☐ 1. Yes, sometimes ☐ 2. Yes, regularly

I.CSID.13 Does he/she forget what happened the day before? If YES, how often does that happen?

☐ 0. No ☐ 1. Yes, sometimes ☐ 2. Yes, regularly

I.CSID.14 Does he/she forget where he/she is? If YES, how often does that happen?

☐ 0. No ☐ 1. Yes, sometimes ☐ 2. Yes, regularly

I.CSID.15 Does he/she get lost in the community? If YES, how often does that happen?

☐ 0. No ☐ 1. Yes, sometimes ☐ 2. Yes, regularly

I.CSID.16 Does he/she get lost at home, e.g. finding the toilet? If YES, how often does that happen?

☐ 0. No ☐ 1. Yes, sometimes ☐ 2. Yes, regularly

I.CSID.17 Does he/she have difficulty performing household chores that he/she used to do, such as preparing food or boiling a pot of tea? If YES, how often does that happen?

☐ 0. No ☐ 1. Yes, sometimes ☐ 2. Yes, regularly

I.CSID.17a Does the INTERVIEWER think that the problem is primarily due to physical disability?

☐ 0. No, not due to physical disability

☐ 1. Yes, due to physical disability

I.CSID.18 Has there been a loss of special skill or hobby he/she could manage before?

☐ 0. No ☐ 1. Yes

I.CSID.19 Has there been a change in his/her ability to handle money?

☐ 0. No, difficulty ☐ 1. Some difficulty

☐ 2. Cannot handle money

I.CSID.20 Does he/she have difficulty in adjusting to change in his/her daily routine? If YES, how often does that happen?

☐ 0. No ☐ 1. Yes, sometimes ☐ 2. Yes, regularly

I.CSID.21 Have you noticed a change in his/her ability to think and reason?

☐ 0. No ☐ 1. Yes

I.CSID.22.1 Does he/she have difficulty feeding himself/herself?

☐ 0. Eats cleanly with proper utensils
☐ 1. Eats messily with a spoon only
☐ 2. Simple solids such as biscuits
☐ 3. Has to be fed

I.CSID.22.2 Does the INTERVIEWER think that the problem is primarily due to physical disability?

☐ 0. No, not due to physical disability
☐ 1. Yes, due to physical disability

I.CSID.23.1 Does he/she have difficulty dressing?

☐ 0. Dresses self
☐ 1. Occasionally misplaces buttons etc.
☐ 2. Wrong sequences, commonly forgets items
☐ 3. Unable to dress

I.CSID.23.2 Does the Interviewer think that the problem is primarily due to physical disability?

☐ 0. No, not due to physical disability
☐ 1. Yes, due to physical disability

I.CSID.24.1 Does he/she have difficulty using the toilet? Does he/she wet or soil himself/herself?

☐ 0. No problems ☐ 1. Occasionally wets bed
☐ 2. Frequently wets bed ☐ 3. Double incontinence

I.CSID.24.2 Does the Interviewer think that the problem is primarily due to physical disability?

☐ 0. No, not due to physical disability
☐ 1. Yes, due to physical disability

I.CSID.25 Does he/she mistake you (or other family or friends) for someone else?

☐ 0. No ☐ 1. Yes

I.CSID.26 Has he/she found difficulty in making decisions recently about everyday things?

☐ 0. No ☐ 1. Yes

I.CSID.27 Does his/her thinking ever seem muddled?

☐ 0. No ☐ 1. Yes

INTERVIEWER CONFIDENCE IN DATA

I.HAS.54 OVERALL RATING OF CONFIDENCE IN DATA

☐ 0. Reasonable ☐ 1. A few doubts ☐ 2. Moderate doubts ☐ 3. Grave doubts ☐ 4. Worthless

(REVISE IF NECESSARY AFTER COMPLETING MODULE 4)

Section 3 - SOCIO-ECONOMIC IMPACT OF DEMENTIA IN TRINIDAD AND TOBAGO

LISTING OF HOUSEHOLD MEMBERS AND EXTERNAL FINANCIAL CONTRIBUTORS

Individual No.	Surname	First Name	Age	Does person reside within the home?
01				☐ 1. Yes ☐ 2. No
02				☐ 1. Yes ☐ 2. No
03				☐ 1. Yes ☐ 2. No
04				☐ 1. Yes ☐ 2. No
05				☐ 1. Yes ☐ 2. No
06				☐ 1. Yes ☐ 2. No
07				☐ 1. Yes ☐ 2. No
08				☐ 1. Yes ☐ 2. No
09				☐ 1. Yes ☐ 2. No
10				☐ 1. Yes ☐ 2. No
11				☐ 1. Yes ☐ 2. No
12				☐ 1. Yes ☐ 2. No

MARK CHOICE BOXES LIKE THIS ☒

1: HOUSEHOLD COMPOSITION AND ECONOMIC ACTIVITY

This section relates to information regarding the FINANCIAL CONTRIBUTORS TO THE HOUSEHOLD.

**1.1 Individual No.**	**1.2 Names of FINANCIAL contributors to the household. (Include local internal, local external and abroad contributors.)**<br><br>What are the names of ALL persons who financially support the houshold?	**1.3 Live-ins**<br><br>Where does N live?<br><br>*1. Local: Internal*<br>*2. Local: External*<br>*3. Abroad*	**1.4 Age**<br><br>What was N's age at last birthday?<br>**(Insert 'date of birth' in space provided in the format of dd/mm/yy)**	**1.5 Sex**<br><br>What is N's sex?<br><br>*1. Male*<br>*2. Female*	**1.6 Relationship to Head of Household**<br><br>What is the relationship of N to the head of the household?<br>*1. Head*<br>*2. Spouse/Partner*<br>*3. Child*<br>*4. Parent*<br>*5. Other relative*<br>*6. Non relative*<br>*99. Not stated*
☐☐		☐ 1 ☐ 2 ☐ 3	☐☐☐ *years*<br>________<br>☐ 98. Don't know	☐ 1 ☐ 2	☐ 1 ☐ 2 ☐ 3 ☐ 4<br>☐ 5 ☐ 6 ☐ 99
☐☐		☐ 1 ☐ 2 ☐ 3	☐☐☐ *years*<br>________<br>☐ 98. Don't know	☐ 1 ☐ 2	☐ 1 ☐ 2 ☐ 3 ☐ 4<br>☐ 5 ☐ 6 ☐ 99
☐☐		☐ 1 ☐ 2 ☐ 3	☐☐☐ *years*<br>________<br>☐ 98. Don't know	☐ 1 ☐ 2	☐ 1 ☐ 2 ☐ 3 ☐ 4<br>☐ 5 ☐ 6 ☐ 99
☐☐		☐ 1 ☐ 2 ☐ 3	☐☐☐ *years*<br>________<br>☐ 98. Don't know	☐ 1 ☐ 2	☐ 1 ☐ 2 ☐ 3 ☐ 4<br>☐ 5 ☐ 6 ☐ 99
☐☐		☐ 1 ☐ 2 ☐ 3	☐☐☐ *years*<br>________<br>☐ 98. Don't know	☐ 1 ☐ 2	☐ 1 ☐ 2 ☐ 3 ☐ 4<br>☐ 5 ☐ 6 ☐ 99
☐☐		☐ 1 ☐ 2 ☐ 3	☐☐☐ *years*<br>________<br>☐ 98. Don't know	☐ 1 ☐ 2	☐ 1 ☐ 2 ☐ 3 ☐ 4<br>☐ 5 ☐ 6 ☐ 99

MARK CHOICE BOXES LIKE THIS ☒

1: HOUSEHOLD COMPOSITION AND ECONOMIC ACTIVITY

Individual No.	**1.7 Ethnic Group**<br><br>To which ethnic group does N belong?<br><br>*1. African*<br>*2. East Indian*<br>*3. Chinese*<br>*4. Syrian/Lebanese*<br>*5. Caucasian*<br>*6. Mixed*<br>*7. Other*<br>*98. Don't Know*<br>*99. Not stated*	**1.8 Education**<br><br>What is N's **highest** level of educational attainment?<br><br>*1. None*<br>*2. Pre-school*<br>*3. Primary*<br>*4. School Leaving*<br>*5. Vocational School*<br>*6. Secondary*<br>*7. Tertiary/Univ*<br>*8. Other*<br>*98. Don't Know*<br>*99. Not stated*	**1.9 Marital/Union Status**<br><br>What is N's marital/union status?<br><br>*1. Never married*<br>*2. Married and living with spouse*<br>*3. Married and not living with spouse*<br>*4. Never had a spouse nor common-law partner*<br>*5. Widowed*<br>*6. Common-law*<br>*7. No longer living with common-law partner*<br>*8. Divorced*<br>*9. Legally separated*<br>*10. Visiting partner*<br>*11. Other*<br>*98. Don't know*<br>*99. Not stated*	**1.10 Type of Worker**<br>What is N's occupation?<br><br>**(If unemployed, retired or over 60, SKIP TO Q. 1.13)**<br><br>*1. Agricultural Worker (self-employed)*<br>*2. Agricultural Worker (working for others)*<br>*3. Associate Professional (technical, nursing, artistic)*<br>*4. Clerical Worker/Secretary*<br>*5. Housewife*<br>*6. Manager/Administrator*<br>*7. Professional (health, teaching, legal, financial)*<br>*8. Semi-skilled Labourer (helper of skilled labourer)*<br>*9. Skilled Labourer (building, electrical etc)*<br>*10. Unskilled Labourer*<br>*98. Don't know*<br>*99. Not stated*
☐☐	☐ 1 ☐ 2 ☐ 3<br>☐ 4 ☐ 5 ☐ 6<br>☐ 7 ☐ 98 ☐ 99	☐ 1 ☐ 2 ☐ 3<br>☐ 4 ☐ 5 ☐ 6<br>☐ 7 ☐ 8 ☐ 98<br>☐ 99	☐ 1 ☐ 2 ☐ 3 ☐ 4 ☐ 5<br>☐ 6 ☐ 7 ☐ 8 ☐ 9 ☐ 10<br>☐ 11 ☐ 98 ☐ 99	☐ 1 ☐ 2 ☐ 3 ☐ 4<br>☐ 5 ☐ 6 ☐ 7 ☐ 8<br>☐ 9 ☐ 10 ☐ 98 ☐ 99
☐☐	☐ 1 ☐ 2 ☐ 3<br>☐ 4 ☐ 5 ☐ 6<br>☐ 7 ☐ 98 ☐ 99	☐ 1 ☐ 2 ☐ 3<br>☐ 4 ☐ 5 ☐ 6<br>☐ 7 ☐ 8 ☐ 98<br>☐ 99	☐ 1 ☐ 2 ☐ 3 ☐ 4 ☐ 5<br>☐ 6 ☐ 7 ☐ 8 ☐ 9 ☐ 10<br>☐ 11 ☐ 98 ☐ 99	☐ 1 ☐ 2 ☐ 3 ☐ 4<br>☐ 5 ☐ 6 ☐ 7 ☐ 8<br>☐ 9 ☐ 10 ☐ 98 ☐ 99
☐☐	☐ 1 ☐ 2 ☐ 3<br>☐ 4 ☐ 5 ☐ 6<br>☐ 7 ☐ 98 ☐ 99	☐ 1 ☐ 2 ☐ 3<br>☐ 4 ☐ 5 ☐ 6<br>☐ 7 ☐ 8 ☐ 98<br>☐ 99	☐ 1 ☐ 2 ☐ 3 ☐ 4 ☐ 5<br>☐ 6 ☐ 7 ☐ 8 ☐ 9 ☐ 10<br>☐ 11 ☐ 98 ☐ 99	☐ 1 ☐ 2 ☐ 3 ☐ 4<br>☐ 5 ☐ 6 ☐ 7 ☐ 8<br>☐ 9 ☐ 10 ☐ 98 ☐ 99
☐☐	☐ 1 ☐ 2 ☐ 3<br>☐ 4 ☐ 5 ☐ 6<br>☐ 7 ☐ 98 ☐ 99	☐ 1 ☐ 2 ☐ 3<br>☐ 4 ☐ 5 ☐ 6<br>☐ 7 ☐ 8 ☐ 98<br>☐ 99	☐ 1 ☐ 2 ☐ 3 ☐ 4 ☐ 5<br>☐ 6 ☐ 7 ☐ 8 ☐ 9 ☐ 10<br>☐ 11 ☐ 98 ☐ 99	☐ 1 ☐ 2 ☐ 3 ☐ 4<br>☐ 5 ☐ 6 ☐ 7 ☐ 8<br>☐ 9 ☐ 10 ☐ 98 ☐ 99
☐☐	☐ 1 ☐ 2 ☐ 3<br>☐ 4 ☐ 5 ☐ 6<br>☐ 7 ☐ 98 ☐ 99	☐ 1 ☐ 2 ☐ 3<br>☐ 4 ☐ 5 ☐ 6<br>☐ 7 ☐ 8 ☐ 98<br>☐ 99	☐ 1 ☐ 2 ☐ 3 ☐ 4 ☐ 5<br>☐ 6 ☐ 7 ☐ 8 ☐ 9 ☐ 10<br>☐ 11 ☐ 98 ☐ 99	☐ 1 ☐ 2 ☐ 3 ☐ 4<br>☐ 5 ☐ 6 ☐ 7 ☐ 8<br>☐ 9 ☐ 10 ☐ 98 ☐ 99
☐☐	☐ 1 ☐ 2 ☐ 3<br>☐ 4 ☐ 5 ☐ 6<br>☐ 7 ☐ 98 ☐ 99	☐ 1 ☐ 2 ☐ 3<br>☐ 4 ☐ 5 ☐ 6<br>☐ 7 ☐ 8 ☐ 98<br>☐ 99	☐ 1 ☐ 2 ☐ 3 ☐ 4 ☐ 5<br>☐ 6 ☐ 7 ☐ 8 ☐ 9 ☐ 10<br>☐ 11 ☐ 98 ☐ 99	☐ 1 ☐ 2 ☐ 3 ☐ 4<br>☐ 5 ☐ 6 ☐ 7 ☐ 8<br>☐ 9 ☐ 10 ☐ 98 ☐ 99

1: HOUSEHOLD COMPOSITION AND ECONOMIC ACTIVITY

Individual Number	1.11 Main Employment Status<br><br>What was N's employment status **LAST WEEK**?<br><br>*1. Permanent*<br>*2. Temporary*<br>*3. Contract*<br>*4. Seasonal*<br>*5. Occasional/Odd Jobs*<br>*6. Not applicable*<br>*98. Don't know*<br>*99. Not stated*	1.12 Work Income<br><br>What is N's gross **MONTHLY AVERAGE INCOME** from employment to the nearest $?<br><br>*1. Less than TT$ 2,000*<br>*2. TT$ 2,001-TT$ 4,000*<br>*3. TT$ 4,001-TT$ 7,000*<br>*4. More than TT$ 7,001*<br>*5. Not applicable*<br>*98. Don't know*<br>*99. Not stated*	1.13 Value of Remittances<br><br>What is the **MONTHLY AVERAGE** amount that N sends/receives from abroad?<br><br>*1. Less than US$ 100 (TT$ 600)*<br>*2. US$ 101-US$ 200 (TT$601-TT$1,200)*<br>*3. More than US$ 201 (TT$ 1,200)*<br>*4. None*<br>*98. Don't know*<br>*99. Not stated*	1.14 NIS<br><br>Does N receive NIS?<br><br>**(Only for persons 60 years and older or Retirees)**<br><br>*1. Yes*<br>*2. No*<br>*98. Don't know*<br>*99. Not stated*	1.15 Value of NIS<br>**(Only if YES to Q1.14)**<br><br>How much does N receive **MONTHLY**? **(to the nearest dollar)**<br><br>**(Only for persons 60 years and older or Retirees)**
☐☐	☐ 1 ☐ 2<br>☐ 3 ☐ 4<br>☐ 5 ☐ 6<br>☐ 98 ☐ 99	☐ 1 ☐ 2 ☐ 3<br>☐ 4 ☐ 5 ☐ 98<br>☐ 99	☐ 1 ☐ 2 ☐ 3<br>☐ 4 ☐ 98 ☐ 99	☐ 1 ☐ 2<br>☐ 98 ☐ 99	☐☐☐☐☐☐
☐☐	☐ 1 ☐ 2<br>☐ 3 ☐ 4<br>☐ 5 ☐ 6<br>☐ 98 ☐ 99	☐ 1 ☐ 2 ☐ 3<br>☐ 4 ☐ 5 ☐ 98<br>☐ 99	☐ 1 ☐ 2 ☐ 3<br>☐ 4 ☐ 98 ☐ 99	☐ 1 ☐ 2<br>☐ 98 ☐ 99	☐☐☐☐☐☐
☐☐	☐ 1 ☐ 2<br>☐ 3 ☐ 4<br>☐ 5 ☐ 6<br>☐ 98 ☐ 99	☐ 1 ☐ 2 ☐ 3<br>☐ 4 ☐ 5 ☐ 98<br>☐ 99	☐ 1 ☐ 2 ☐ 3<br>☐ 4 ☐ 98 ☐ 99	☐ 1 ☐ 2<br>☐ 98 ☐ 99	☐☐☐☐☐☐
☐☐	☐ 1 ☐ 2<br>☐ 3 ☐ 4<br>☐ 5 ☐ 6<br>☐ 98 ☐ 99	☐ 1 ☐ 2 ☐ 3<br>☐ 4 ☐ 5 ☐ 98<br>☐ 99	☐ 1 ☐ 2 ☐ 3<br>☐ 4 ☐ 98 ☐ 99	☐ 1 ☐ 2<br>☐ 98 ☐ 99	☐☐☐☐☐☐
☐☐	☐ 1 ☐ 2<br>☐ 3 ☐ 4<br>☐ 5 ☐ 6<br>☐ 98 ☐ 99	☐ 1 ☐ 2 ☐ 3<br>☐ 4 ☐ 5 ☐ 98<br>☐ 99	☐ 1 ☐ 2 ☐ 3<br>☐ 4 ☐ 98 ☐ 99	☐ 1 ☐ 2<br>☐ 98 ☐ 99	☐☐☐☐☐☐
☐☐	☐ 1 ☐ 2<br>☐ 3 ☐ 4<br>☐ 5 ☐ 6<br>☐ 98 ☐ 99	☐ 1 ☐ 2 ☐ 3<br>☐ 4 ☐ 5 ☐ 98<br>☐ 99	☐ 1 ☐ 2 ☐ 3<br>☐ 4 ☐ 98 ☐ 99	☐ 1 ☐ 2<br>☐ 98 ☐ 99	☐☐☐☐☐☐

2: ACCESS TO SOCIAL PROGRAMMES

This Section applies to ALL HOUSEHOLD MEMBERS, INCLUDING CHILDREN.

2.2 GOVERNMENT SOCIAL PROGRAMMES

**2.1 Please state which household members access social programmes.**<br><br>**INDIVIDUAL NUMBER**	**2.2 Social Programmes**<br><br>What type(s) of programme(s) do(es) N currently access? **(MULTIPLE RESPONSES ALLOWED)**<br><br>*1. Old Age Pension*<br>*2. Public Assistance*<br>*3. Disability Grant*<br>*4. CDAP*<br>*5. ARV Treatment*<br>*6. GATE*<br>*7. Smart Card*<br>*8. School Meals*<br>*9. Other (specify)*
☐☐	☐ 1 ☐ 2 ☐ 3 ☐ 4 ☐ 5 ☐ 6 ☐ 7 ☐ 8<br>☐ 9 ______________
☐☐	☐ 1 ☐ 2 ☐ 3 ☐ 4 ☐ 5 ☐ 6 ☐ 7 ☐ 8<br>☐ 9 ______________
☐☐	☐ 1 ☐ 2 ☐ 3 ☐ 4 ☐ 5 ☐ 6 ☐ 7 ☐ 8<br>☐ 9 ______________
☐☐	☐ 1 ☐ 2 ☐ 3 ☐ 4 ☐ 5 ☐ 6 ☐ 7 ☐ 8<br>☐ 9 ______________
☐☐	☐ 1 ☐ 2 ☐ 3 ☐ 4 ☐ 5 ☐ 6 ☐ 7 ☐ 8<br>☐ 9 ______________
☐☐	☐ 1 ☐ 2 ☐ 3 ☐ 4 ☐ 5 ☐ 6 ☐ 7 ☐ 8<br>☐ 9 ______________

MARK CHOICE BOXES LIKE THIS ☒

3: MORBIDITY

IDENTIFICATION

3.1 Individual number of elderly person selected for the study. ☐☐ **First Name:** ____________________

DIAGNOSIS

3.2a Was your relative diagnosed with any of the following illness(es)? (MULTIPLE RESPONSES ALLOWED)

☐ 1. Angina	☐ 2. Alzheimer's Disease	☐ 3. Arthritis	☐ 4. Asthma
☐ 5. Cancer	☐ 6. Dementia	☐ 7. Depression	☐ 8. Diabetes
☐ 9. Epilepsy	☐ 10. Head Injury	☐ 11. Heart Disease	☐ 12. High Cholesterol
☐ 13. Hypertension	☐ 14. Parkinson's Disease	☐ 15. Stroke	☐ 16. None
☐ 17. Other (specify)	____________________	☐ 98. Don't know	☐ 99. Not stated

3.2b Please indicate medications used and their cost per month? (Include drugs covered under the CDAP Programme.) (ROUND TO THE NEAREST DOLLAR)

MEDICATION USED	TOTAL COST (PER MONTH)
1. Medication for dementia	☐☐☐☐☐☐
2. Medication for high blood pressure	☐☐☐☐☐☐
3. Aspirin	☐☐☐☐☐☐
4. Medication to thin blood	☐☐☐☐☐☐
5. Medication for diabetes/sugar	☐☐☐☐☐☐
6. Insulin	☐☐☐☐☐☐
7. Medication for pain	☐☐☐☐☐☐
8. Medication for high cholesterol	☐☐☐☐☐☐
9. Vitamins	☐☐☐☐☐☐
10. Other (specify) ____________________	☐☐☐☐☐☐

3: MORBIDITY

	Persistent Sadness	Memory Loss
INPATIENT VISITS<br>***Admission***<br>**3.3 Has your relative been admitted into the hospital in the LAST YEAR because of:**	☐ 1. Yes **(GO TO Q. 3.4)**<br>☐ 2. No **(SKIP TO Q. 3.8)**<br>☐ 98. Don't know **(SKIP TO Q. 3.8)**<br>☐ 99. Not stated **(SKIP TO Q. 3.8)**	☐ 1. Yes **(GO TO Q. 3.4)**<br>☐ 2. No **(SKIP TO Q. 3.8)**<br>☐ 98. Don't know **(SKIP TO Q. 3.8)**<br>☐ 99. Not stated **(SKIP TO Q. 3.8)**
TYPE OF INSTITUTION<br>**3.4 On the LAST occasion, was your relative admitted into a private or public hospital?**	☐ 1. Private **(GO TO Q. 3.5)**<br>☐ 2. Public **(SKIP TO Q. 3.6)**<br>☐ 98. Don't know **(SKIP TO Q. 3.6)**<br>☐ 99. Not stated **(SKIP TO Q. 3.6)**	☐ 1. Private **(GO TO Q. 3.5)**<br>☐ 2. Public **(SKIP TO Q. 3.6)**<br>☐ 98. Don't know **(SKIP TO Q. 3.6)**<br>☐ 99. Not stated **(SKIP TO Q. 3.6)**
**HOSPITAL CHARGE**<br>**3.5 On this LAST occasion, what was the hospital charge? (ROUND TO THE NEAREST DOLLAR)**	$ ☐☐☐,☐☐☐<br>☐ 98. Don't know ☐ 99. Not stated	$ ☐☐☐,☐☐☐<br>☐ 98. Don't know ☐ 99. Not stated
LENGTH OF STAY<br>**3.6 On this LAST occasion, how long did your relative stay in the hospital?**	☐ 1. Less than one day<br>☐ 2. One-four days<br>☐ 3. More than four days<br>☐ 98. Don't know<br>☐ 99. Not stated	☐ 1. Less than one day<br>☐ 2. One-four days<br>☐ 3. More than four days<br>☐ 98. Don't know<br>☐ 99. Not stated
TRANSPORT COST<br>**3.7 What were the transportation costs on the LAST occasion? (ROUND TO THE NEAREST DOLLAR)**	$ ☐☐,☐☐☐<br>☐ 1. None ☐ 98. Don't know<br>☐ 99. Not stated	$ ☐☐,☐☐☐<br>☐ 1. None ☐ 98. Don't know<br>☐ 99. Not stated
OUTPATIENT VISITS<br>***Frequency***<br>**3.8 How many times in the LAST YEAR has your relative visited a doctor/had a doctor visit as an outpatient?**	☐ 1. 0 times **(SKIP TO Q. 3.12)**<br>☐ 2. One time<br>☐ 3. Two-three times<br>☐ 4. Four-five times<br>☐ 5. Six or more times<br>☐ 98. Don't know **(SKIP TO Q. 3.12)**<br>☐ 99. Not stated **(SKIP TO Q. 3.12)**	☐ 1. 0 times **(SKIP TO Q. 3.12)**<br>☐ 2. One time<br>☐ 3. Two-three times<br>☐ 4. Four-five times<br>☐ 5. Six or more times<br>☐ 98. Don't know **(SKIP TO Q. 3.12)**<br>☐ 99. Not stated **(SKIP TO Q. 3.12)**
TYPE<br>**3.9 Was the doctor a generalized doctor or a specialized doctor?**	☐ 1. Generalized Doctor<br>☐ 2. Specialized Doctor<br>☐ 98. Don't know<br>☐ 99. Not stated	☐ 1. Generalized Doctor<br>☐ 2. Specialized Doctor<br>☐ 98. Don't know<br>☐ 99. Not stated
COST<br>**3.10 What was the cost PER visit? (ROUND TO THE NEAREST DOLLAR)**<br>***If the visit was "public", then mark as 1. None.***	$ ☐☐,☐☐☐<br>☐ 1. None ☐ 98. Don't know<br>☐ 99. Not stated	$ ☐☐,☐☐☐<br>☐ 1. None ☐ 98. Don't know<br>☐ 99. Not stated
TRANSPORT COST<br>**3.11 On the LAST occasion, what were the transportation costs? (ROUND TO THE NEAREST DOLLAR)**	$ ☐☐,☐☐☐<br>☐ 1. None ☐ 98. Don't know<br>☐ 99. Not stated	$ ☐☐,☐☐☐<br>☐ 1. None ☐ 98. Don't know<br>☐ 99. Not stated

3: MORBIDITY

PURCHASES

3.12 In the PAST YEAR, what special items were purchased to make your relative's life easier? (MULTIPLE RESPONSES ALLOWED) (Please provide the cost incurred in each case.)

ITEMS	COST (in the last year)		COST (in the last year)
☐ 1. Walkers	$ ☐☐☐,☐☐☐	☐ 2. Adult diapers	$ ☐☐☐,☐☐☐
☐ 3. Grab bars/rails	$ ☐☐☐,☐☐☐	☐ 4. Other (specify) ____________	$ ☐☐☐,☐☐☐

☐ 5. None ☐ 98. Don't know ☐ 99. Not stated

PROCEDURES

Type

3.13 Has your relative had any of the following procedures done in the PAST 5 YEARS? (MULTIPLE RESPONSES ALLOWED) (Please provide cost incurred in each case. If procedure was done "publicly" then mark cost as "0".)

PROCEDURE	COST		
☐ 1. EKG/ECG	$ ☐☐☐,☐☐☐	☐ 2. CT/MRI	$ ☐☐☐,☐☐☐
☐ 3. X-Ray	$ ☐☐☐,☐☐☐	☐ 4. EEG	$ ☐☐☐,☐☐☐
☐ 5. Other (specify) ____________			$ ☐☐☐,☐☐☐

☐ 6. None ☐ 98. Don't know ☐ 99. Not stated

ACCIDENTS

3.14 Does your relative drive?

☐ 1. Yes **(GO TO Q. 3.15)** ☐ 2. No **(SKIP TO Q. 4.1)** ☐ 98. Don't know **(SKIP TO Q. 4.1)** ☐ 99. Not stated **(SKIP TO Q. 4.1)**

3.15 As a driver, has he/she had any vehicular accidents in the PAST YEAR?

☐ 1. Yes **(GO TO Q. 3.16)** ☐ 2. No **(SKIP TO Q. 4.1)** ☐ 98. Don't know **(SKIP TO Q. 4.1)** ☐ 99. Not stated **(SKIP TO Q. 4.1)**

3.16 What was the estimated cost of the MOST RECENT accident in which your relative was involved?

$ ☐☐☐,☐☐☐

4: ASSETS

4.1 What type of stove does your household use MOST? (SINGLE RESPONSE)

☐ 1. Gas stove ☐ 2. Electric stove ☐ 3. Coal ☐ 4. Kerosene

4.2 What other kitchen item(s) do(es) your household have? (MULTIPLE RESPONSES ALLOWED)

☐ 1. Microwave oven ☐ 2. Refrigerator ☐ 3. Deep freezer

4.3 What is the MAIN source of lighting in your dwelling? (SINGLE RESPONSE)

☐ 1. Electricity ☐ 2. Oil/Kerosene ☐ 3. No lighting

4.4 What entertainment item(s) is/are in your household? (MULTIPLE RESPONSES ALLOWED)

☐ 1. Radio ☐ 2. Television*

☐ 3. DVD player ☐ 4. Game console

SKIP TO Q. 4.6 IF THE HOUSEHOLD DOES NOT HAVE A TV.

4.5 What TV facility does the household have? (SINGLE RESPONSE)

☐ 1. Direct TV ☐ 2. Cable TV

☐ 3. Basic TV

4.6 What telephone facility(ies) do(es) your household have? (MULTIPLE RESPONSES ALLOWED)

☐ 1. Telephone-fixed line ☐ 2. Mobile phone

4.7 Does anyone in your household have a personal computer?

☐ 1. Yes **(GO TO Q. 4.8)** ☐ 2. No **(SKIP TO Q. 5.1)**

☐ 98. Don't know **(SKIP TO Q. 5.1)**

4.8 Does your household have access to the Internet?

☐ 1. Yes ☐ 2. No ☐ 98. Don't know

5: SAVINGS

5.1 Does the household have any form(s) of savings?

☐ 1. Yes **(GO TO Q. 5.2)** ☐ 2. No **(GO TO Q. 5.3)**

☐ 98. Don't know **(GO TO Q. 5.3)**

☐ 99. Not stated **(GO TO Q. 5.3)**

5.2 What different forms of savings do/es the household have? (MULTIPLE RESPONSES ALLOWED)

☐ 1. Bank or Credit Union ☐ 2. Unit trusts or shares

☐ 3. Retirement annuity ☐ 4. Life insurance

☐ 5. Funeral policy

☐ 6. Informal saving associations (e.g. Sou Sou)

☐ 7. Other (specify) ____________________

☐ 98. Don't know

☐ 99 Not stated

5.3 Does the health of your relative reduce the household's ability to save?

☐ 1. Yes **(GO TO Q. 5.4)**

☐ 2. No **(SKIP TO Q. 6.1)**

☐ 98. Don't know **(SKIP TO Q. 6.1)**

☐ 99. Not stated **(SKIP TO Q. 6.1)**

5.4 In what way(s)?

____

____

____

6: HOUSEHOLD DEBT

6.1 Does the household currently have any outstanding debts?

☐ 1. Yes **(GO TO Q. 6.2)** ☐ 2. No **(SKIP TO Q. 7.1)**

☐ 98. Don't know **(SKIP TO Q. 7.1)**

☐ 99. Not stated **(SKIP TO Q. 7.1)**

6.2 What type(s) of debt does the household have? (MULTIPLE RESPONSES ALLOWED)

☐ 1. Mortgage ☐ 2. Short term bank loan

☐ 3. Moneys owed to individuals

☐ 4. Other (specify) ____________________

☐ 98. Don't know

☐ 99. Not stated

6.3 Does the health of your relative contribute to the overall debt of the household?

☐ 1. Yes **(GO TO Q. 6.4)**

☐ 2. No **(SKIP TO Q. 7.1)**

☐ 98. Don't know **(SKIP TO Q. 7.1)**

☐ 99. Not stated **(SKIP TO Q. 7.1)**

6.4 In what way(s)?

____

____

____

7: HOUSEHOLD RESPONSES TO FINANCIAL CRISES

7.1 During the LAST 12 MONTHS, did the household experience any financial crisis whereby cash in hand could not cover expenses because of the elderly person's health?

☐ 1. Yes **(GO TO Q. 7.2)** ☐ 2. No **(SKIP TO Q. 8.1)**

☐ 98. Don't know **(SKIP TO Q. 8.1)**

☐ 99. Not stated **(SKIP TO Q. 8.1)**

7.2 How did the household respond financially to such a situation? (MULTIPLE RESPONSES ALLOWED)

☐ 1. Utilized savings **(ASK ONLY Q. 7.3)**

☐ 2. Borrowed money **(ASK ONLY Q. 7.4 and 7.5)**

☐ 3. Sold assets **(ASK ONLY Q. 7.6 and 7.7)**

7.3 What was the PRIMARY purpose of utilizing savings? (SINGLE RESPONSE)

☐ 1. Food ☐ 2. Education ☐ 3. Durables

☐ 4. Medical expenses ☐ 5. Funeral

☐ 6. Other (specify) ____________________

7.4 What was the PRIMARY purpose for borrowing money? (SINGLE RESPONSE)

☐ 1. Food ☐ 2. Education ☐ 3. Durables

☐ 4. Medical expenses ☐ 5. Funeral

☐ 6. Other (specify) ____________________

7.5 From whom/where did your household borrow this money? (SINGLE RESPONSE)

☐ 1. Relative/friend ☐ 2. Employer ☐ 3. Bank

☐ 4. Money Lender ☐ 5. SouSou ☐ 6. Gov't Agency

☐ 7. Landlord ☐ 8. Shopkeeper ☐ 9. Church

☐ 10. Other (specify) ____________________

7.6 What was the PRIMARY purpose of selling assets? (SINGLE RESPONSE)

☐ 1. Food ☐ 2. Education ☐ 3. Durables

☐ 4. Medical expenses ☐ 5. Funeral

☐ 6. Other (specify) ____________________

7.7 What type(s) of assets were sold? (MULTIPLE RESPONSES ALLOWED)

☐ 1. Household appliances ☐ 2. Furniture ☐ 3. Vehicles

☐ 4. Livestock ☐ 5. Household tools ☐ 6. House

☐ 7. Other (specify) ____________________

MARK CHOICE BOXES LIKE THIS ☒

8: MIGRATION

8.1 During the <u>LAST 12 MONTHS</u>, did anyone <u>MOVE OUT</u> because of the elderly perons's health?

☐ 1. Yes **(GO TO Q. 8.2)** ☐ 2. No **(SKIP TO Q. 8.5)** ☐ 98. Don't know **(SKIP TO Q. 8.5)** ☐ 99. Not stated **(SKIP TO Q. 8.5)**

8.2 How many such persons moved out? ☐

8.3 Did this affect the household's ability to care for the elderly person?

☐ 1. Yes **(GO TO Q. 8.4)** ☐ 2. No **(SKIP TO Q. 8.5)** ☐ 98. Don't know **(SKIP TO Q. 8.5)** ☐ 99. Not stated **(SKIP TO Q. 8.5)**

8.4 In what way(s) did the person(s) contribute to the household? <u>(MULTIPLE RESPONSES ALLOWED)</u>

☐ 1. In cash ☐ 2. In kind (specify) ____________ ☐ 3. Caregiving of the elderly person

☐ 98. Don't know ☐ 99. Not stated

8.5 During the <u>LAST 12 MONTHS</u>, did anyone <u>JOIN</u> this household because of the elderly person's health?

☐ 1. Yes **(GO TO Q. 8.6)** ☐ 2. No **(SKIP TO Q. 9.1)** ☐ 98. Don't know **(SKIP TO Q. 9.1)** ☐ 99. Not stated **(SKIP TO Q. 9.1)**

8.6 How many such persons joined? ☐

8.7 In what way(s) did the person(s) contribute to the household? (MULTIPLE RESPONSES ALLOWED)

☐ 1. In cash ☐ 2. In kind (specify) ____________ ☐ 3. Caregiving of the elderly person

☐ 98. Don't know ☐ 99. Not stated

9: CAREGIVING

Note: If the respondent (head of the household) is the elderly person selected for the survey and you are absolutely certain that there is no caregiver, then END the interview.

<u>IDENTIFICATION</u>

9.1 Who is/are the main caregiver(s)? ***The caregiver(s) is/are the person(s) who spend(s) the <u>MOST</u> time (40% of the day) with the elderly person.***

☐ 1. The head of the household (the respondent)

☐ 2. Paid individual(s) **(ASK Q. 9.2, Q. 9.3 and Q. 9.5 ONLY)**

☐ 3. Unpaid individuals **(ASK Q. 9.4 and Q. 9.5 ONLY)**

9.2 How many <u>PAID</u> caregiver(s) are there?

☐ 1. One caregiver ☐ 2. Two caregivers

☐ 3. Three caregivers

☐ 4. Other (specify) ☐

9.3 What is the amount <u>PER MONTH</u> paid to caregiver(s)?

Amount	Caregiver 1	Caregiver 2	Caregiver 3
1. TT$ 500-TT$ 1,500	☐	☐	☐
2. TT$ 1,501-TT$ 2,500	☐	☐	☐
3. TT$ 2,501-TT$ 3,000	☐	☐	☐
4. More than TT$ 3,000	☐	☐	☐
98. Don't know	☐	☐	☐
99. Not stated	☐	☐	☐

9.4 How many <u>UNPAID</u> caregivers are there?

☐ 1. One caregiver ☐ 2. Two caregivers

☐ 3. Three caregivers ☐ 4. Other (specify) ☐

9.5 As the head of the household, indicate how difficult it is to find help for the elderly person?

☐ 1. Not difficult ☐ 2. Somewhat difficult ☐ 3. Very difficult

END OF QUESTIONNAIRE
THANK YOU FOR PARTICIPATING IN OUR HOUSEHOLD SURVEY.
WE ARE VERY GRATEFUL FOR YOUR TIME AND COOPERATION.

47614
